# Transcriptome-Based Network Analysis Reveals a Spectrum Model of Human Macrophage Activation

**DOI:** 10.1016/j.immuni.2014.01.006

**Published:** 2014-02-20

**Authors:** Jia Xue, Susanne V. Schmidt, Jil Sander, Astrid Draffehn, Wolfgang Krebs, Inga Quester, Dominic De Nardo, Trupti D. Gohel, Martina Emde, Lisa Schmidleithner, Hariharasudan Ganesan, Andrea Nino-Castro, Michael R. Mallmann, Larisa Labzin, Heidi Theis, Michael Kraut, Marc Beyer, Eicke Latz, Tom C. Freeman, Thomas Ulas, Joachim L. Schultze

**Affiliations:** 1 Genomics and Immunoregulation, LIMES-Institute, University of Bonn, 53115 Bonn, Germany; 2 Institute of Innate Immunity, University Hospitals, University of Bonn, 53127 Bonn, Germany; 3 Division of Infectious Diseases and Immunology, UMass Medical School, Worcester, MA 01605, USA; 4 German Center of Neurodegenerative Diseases (DZNE), 53127 Bonn, Germany; 5 The Roslin Institute and Royal (Dick) School of Veterinary Studies, University of Edinburgh, Easter Bush, Edinburgh, Midlothian EH25 9RG, Scotland, UK

## Abstract

Macrophage activation is associated with profound transcriptional reprogramming. Although much progress has been made in the understanding of macrophage activation, polarization, and function, the transcriptional programs regulating these processes remain poorly characterized. We stimulated human macrophages with diverse activation signals, acquiring a data set of 299 macrophage transcriptomes. Analysis of this data set revealed a spectrum of macrophage activation states extending the current M1 versus M2-polarization model. Network analyses identified central transcriptional regulators associated with all macrophage activation complemented by regulators related to stimulus-specific programs. Applying these transcriptional programs to human alveolar macrophages from smokers and patients with chronic obstructive pulmonary disease (COPD) revealed an unexpected loss of inflammatory signatures in COPD patients. Finally, by integrating murine data from the ImmGen project we propose a refined, activation-independent core signature for human and murine macrophages. This resource serves as a framework for future research into regulation of macrophage activation in health and disease.

## Highlights

• Macrophages react with specific transcriptional programming upon distinct signals
• Activation by TNF, PGE_2_, and P3C activates a STAT4-associated transcriptional program
• NFKB1, JUNB, and CREB1 are central transcription factors of macrophage activation
• Inflammatory signatures are lost in alveolar macrophages from COPD patients

## Graphical Abstract

**Figure:**
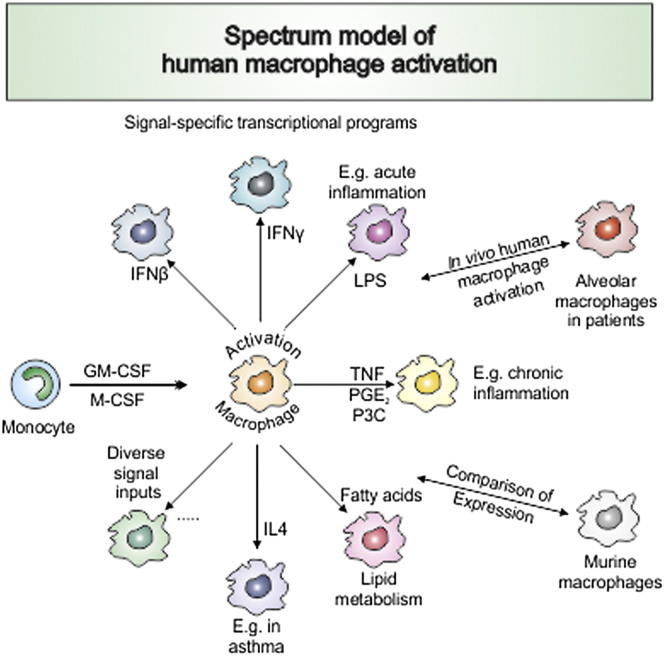

## Introduction

During the last two decades, a conceptual framework for the description of macrophage activation has been developed. According to this framework, macrophages can be polarized into classically (M1) or alternatively (M2) activated cells representing two polar extremes of signals computed by macrophages (Biswas and Mantovani, 2010). The M1 versus M2 model has been very helpful in describing immune responses during acute infections, allergies, asthma, and obesity (Chinetti-Gbaguidi and Staels, 2011). However, observations obtained from macrophages involved in chronic inflammation, chronic infection, or cancer strongly suggest that the myeloid compartment has a much broader transcriptional repertoire depending on the different environmental signals received (Boorsma et al., 2013; Chow et al., 2011; Edin et al., 2012; Hodge et al., 2011; Lawrence and Natoli, 2011; Martinez et al., 2009; Mosser and Edwards, 2008; Murray and Wynn, 2011; Reinartz et al., 2013). Despite a number of genomic studies analyzing macrophage activation in response to bacteria, TLR ligands, and M1 or M2 stimuli, to date there have been no attempts to reconcile these observations by building new and integrative models of macrophage activation (Martinez et al., 2006; McDermott et al., 2011; Nau et al., 2002; Ramsey et al., 2008).

Transcriptomics has considerably contributed to a better understanding of immune cell function and regulation. Large consortia such as the ImmGen consortium (Best et al., 2013; Bezman et al., 2012; Cohen et al., 2013; Gautier et al., 2012; Miller et al., 2012) or the Human Immunology Project Consortium (Poland et al., 2013) compiled extensive data sets and defined a core transcriptional program for murine tissue macrophages and dendritic cells (DCs) under steady-state conditions (Gautier et al., 2012; Miller et al., 2012). A complementary approach has been introduced by InnateDB (Breuer et al., 2013). Data on molecular interactions between proteins of the innate immune system derived from smaller data sets have been compiled and can be used to reveal mechanistic insights into immune cell function (Hume et al., 2010; Mabbott et al., 2010). Unfortunately, meta-analysis of small data sets has been hampered by several challenges, including differences in the genetic background of mice and in stimulation conditions and the combination of in vitro and in vivo data limit or even bias model generation of incongruous data sets (Mabbott et al., 2010). Moreover, comparative studies have identified substantial differences in immune-cell gene expression between mice and humans (Schroder et al., 2012; Shay et al., 2013). Therefore, it remains to be fully elucidated, how immune cell activation—particularly in human macrophages—is transcriptionally controlled and to which degree these pathways are conserved across species (Murray and Wynn, 2011). Standardizing data acquisition and assembling larger data sets, such as by the ImmGen consortium (Heng and Painter, 2008), is necessary to answer such questions.

Several elegant studies have demonstrated the value of analyzing networks based on expression profiling in macrophages (Martinez et al., 2006; Nau et al., 2002; Ramsey et al., 2008) or T helper 17 (Th17) cells (Ciofani et al., 2012; Yosef et al., 2013). These studies show how technological and analytical advances can reveal network structures in immune cells, e.g., by using algorithms that integrate transcriptome data with database-stored information. Other approaches that require large data sets, such as reverse network engineering (RNE), have previously been used to characterize B cell activation (Basso et al., 2005) and have been further refined during the last few years (Marbach et al., 2012). However, so far, RNE has not been applied to other immune cells most likely due to the lack of large enough data sets.

In this study, we generated a resource data set to assess transcriptional regulation during human macrophage activation by comparing a diverse set of stimuli on a single microarray platform under highly standardized conditions. Network modeling of this data set led us to extend the current M1 versus M2 polarization model to a “spectrum model” with at least nine distinct macrophage activation programs. Further characterization of individual programs identified transcription factors associated with particular phenotypes, such as STAT4, which was associated with stimuli linked to chronic inflammation (TNF, prostaglandin E2, TLR2-ligand, “TPP”) as seen in granulomatous diseases (Ciofani et al., 2012; Marino et al., 1997; Reiling et al., 2002; Shay and Celeste Simon, 2012). Furthermore, we applied this resource data set to define activation states of human alveolar tissue macrophages in vivo. Finally, we used our resource data set to refine the previously suggested core macrophage signature to encompass species differences and account for the spectrum of macrophage activation.

## Results

### Extending the Current Model of Macrophage Polarization

**Figure 1:**
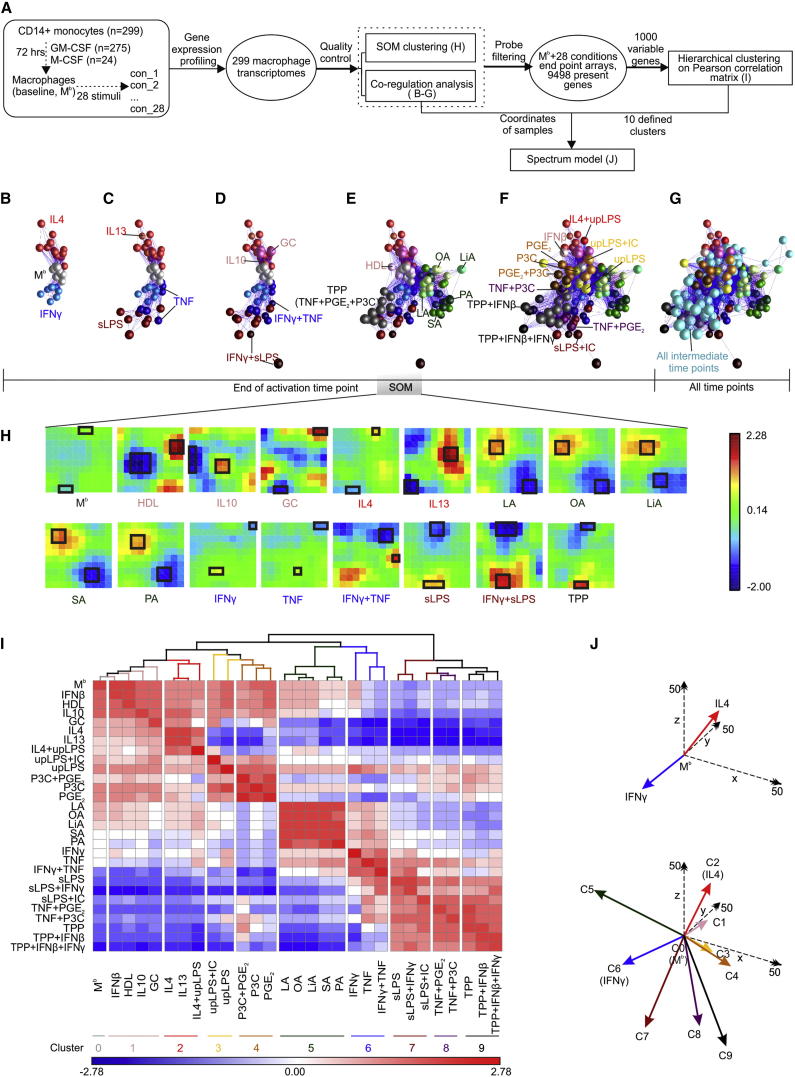
Extending Macrophage Polarization to a Spectrum Model (A) Schema describing the workflow for Figure 1. (B–G) Correlation networks of 299 macrophage transcriptomes representing 29 conditions from end of activation time points (B–F) also including intermediate time points (G). (H) Self-Organizing Map (SOM) clustering using samples displayed in (E). Clusters with the top up- or downregulated genes for each stimulus are marked with a frame. (I) Matrix of hierarchically clustered Pearson’s correlation coefficient matrix (CCM) standardized from −2.78 to 2.78 (blue to red) based on 1,000 most variable probes. (J) Spectrum model (3D) based on the ten clusters defined in (I) and sample values (coordinates) defined by correlation network from (F). Baseline macrophages (M^b^) are set as origin, activation states are represented by colored arrows, x, y, z axes are in dashed lines with double arrows. See also Figure S1, Movie S1, and Table S2.

We generated macrophages from human monocytes by differentiation with GM-CSF or M-CSF (Figure 1A) and compared their transcriptomes with DCs and T, B, and natural killer (NK) cells (see Figure S1A, Table S1, and Supplemental Information available online). With coregulation analysis (CRA) to assess overall sample-to-sample relationships, macrophages were clearly distinguishable from other cell types including DCs (Figures S1B–S1E), which was confirmed on protein level by flow cytometry (Figures S1F and S1G). To better understand the complexity of transcriptional regulation after macrophage activation, we next analyzed the transcriptional programs of macrophages activated with 28 different stimuli including pattern recognition receptor ligands, cytokines, and metabolic cues (Figure 1A). To determine the overall relationship of these activation states within the data set, we first applied CRA (Figures 1B–1G). In agreement with the existing model, a virtual axis was formed, where macrophages at baseline (M^b^) were placed in between macrophages stimulated with interferon-γ (IFN-γ) (M1) and interleukin-4 (IL-4) (M2) (Figure 1B; Movie S1). Adding other conditions linked to M1 (sLPS, TNF) or M2 (IL-13) polarization (Biswas and Mantovani, 2010) (Figure 1C) did not change the overall M1 and M2 axis (Figure 1B). Including further M1- and M2-associated stimuli (IFN-γ+TNF, IL-10) increased the variance in the correlation matrix but the overall bipolar structure was maintained (Figure 1D). However, when adding stimuli not linked to either M1 or M2 polarization, such as free fatty acids, high-density lipoprotein (HDL), or combinations of stimuli associated with chronic inflammation (Table S1), a spectrum of macrophage-activation signatures beyond the initial bipolar axis became apparent (Figures 1E and 1F). Furthermore, when samples generated at earlier time points of stimulation were added, the spectrum of macrophage activation was shown to consist of a dense network of individual signatures (Figure 1G). To bioinformatically validate these findings, we applied Self-Organizing-Map (SOM) clustering (Figure 1H) and correlation coefficient matrices (CCM) (Figure 1I). Performing SOM clustering on the conditions shown by CRA in Figure 1E revealed that every stimulus was characterized by a specific cluster structure (Figure 1H) further supporting the spectrum model. Similarly, we did not identify a bipolar structure within the CCM, but rather a condition-specific spectrum of correlation coefficients in 10 major clusters (Figure 1I). By using the coordinates of the samples defined by CRA within the 10 clusters defined by CCM to build sum vectors in three-dimensional space, we propose a model of macrophage activation best described by a spectrum of transcriptional programs (Figure 1J). Taken together, these data clearly extend the current model of M1 versus M2 polarization to a spectrum model of macrophage activation.

### Identification of Genes Specifically Associated with Distinct Stimuli

**Figure 2:**
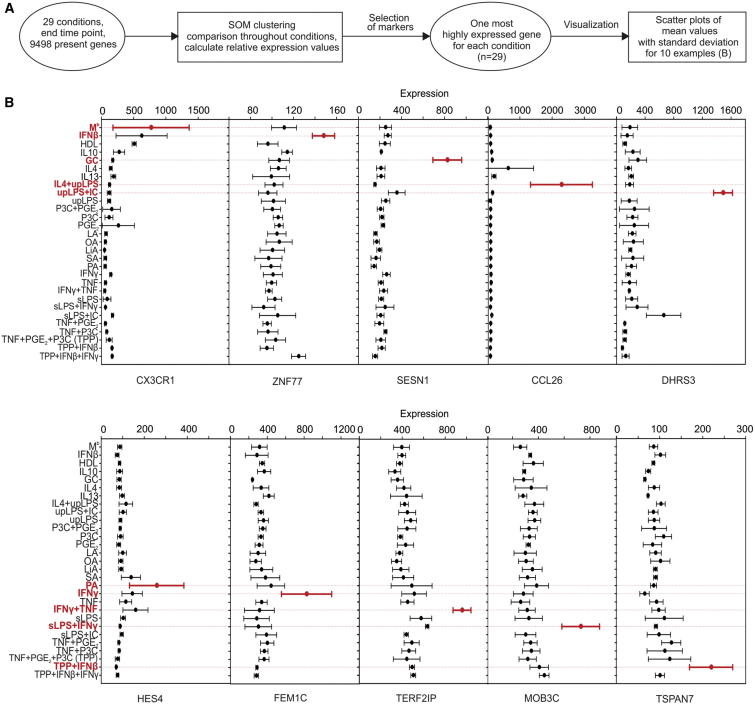
Genes with Selective Expression Associated with Distinct Stimuli (A) Schema describing the workflow for Figure 2. (B) Absolute expression counts (mean ± SD) of genes defined by SOM clustering to be highly expressed for a particular stimulation condition. Shown here are genes enriched in either M^b^ (baseline), IFN-β, GC, IL4+uLPS, upLPS+IC, PA, IFN-γ, IFN-γ +TNF, sLPS+IFN-γ, or TPP+IFN-β. All other conditions are shown in Figure S2.

We next determined whether the different stimuli could be distinguished on the gene level within the complete spectrum of the model. From all macrophage samples included in the analysis, we identified 9,498 genes that were expressed in at least one condition (Table S1D). We used these genes for SOM clustering, allowing us to select genes specifically regulated and enriched for individual stimuli (Figure 2A). In fact, by using this approach we could identify genes that were selectively elevated in only one of the stimulation conditions included in our data set. For example, IFN-β selectively induced *ZNF77*, while IFN-γ selectively induced *FEM1C* (Figure 2B; Figure S2). However, we also found stimuli where a single gene was insufficient to distinguish between closely related conditions, e.g., *SERINC2* was induced by PGE_2_ but also by PGE_2_+P3C, suggesting that gene combinations are necessary to distinguish complex input signals at the transcriptional level. Overall, although some input signals might be associated with the induction of single genes, future studies will require the assessment of a substantial number of markers as surrogates for distinct activation programs of macrophages.

### Network Analysis Defines Stimulus-Associated Programs of Macrophage Activation

**Figure 3:**
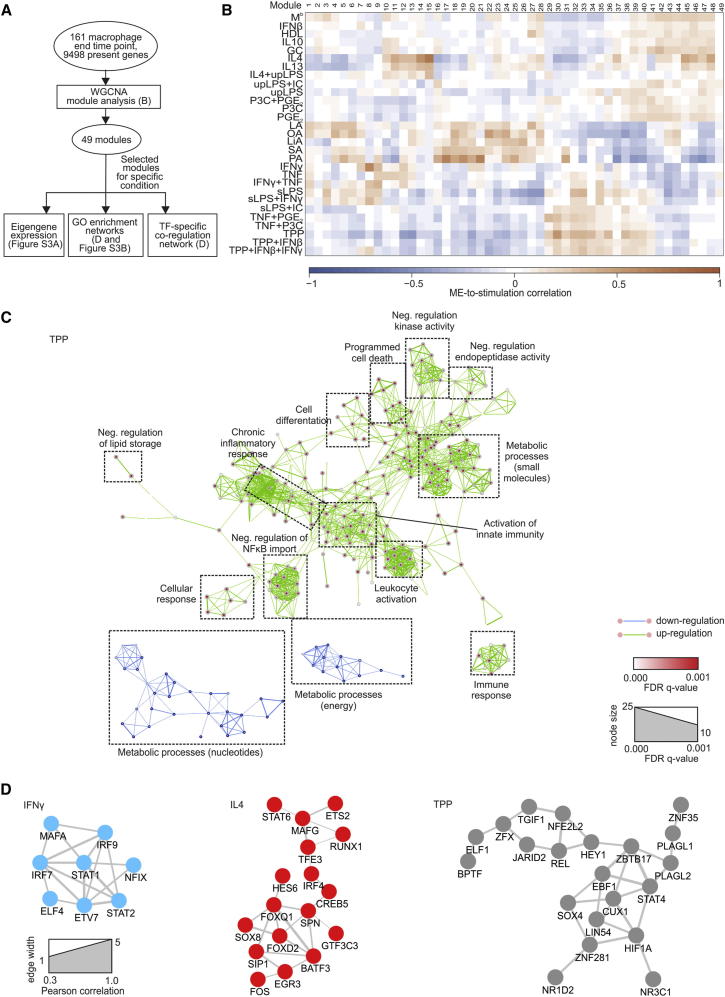
Activation-Specific Genes Revealed by Weighted Correlation Network Analysis (A) Schema describing the workflow for Figure 3. (B) Heatmap showing the correlation of the module eigengene (first principal component; ME) to the traits (stimulation conditions). Blue means negative correlation; orange means positive correlation. (C) Network visualization of GOEA of modules 30, 32–33 (positively correlated), respectively, 12, 13, and 20 (negatively correlated) for TPP-stimulation using BiNGO and EnrichmentMap. Red nodes represent enriched GO-terms, whereas node size represents corresponding FDR-adjusted enrichment p value (q value). Edge thickness shows overlap of genes between neighbor nodes. (D) Correlation network of module-specific transcription factors (TFs) (IFN-γ: modules 7-9; IL-4: modules 13-15; TPP: 30, 32–33). Edge width shows the Pearson’s correlation between each TF pair. See also Figure S3 and Table S2.

To investigate stimulus-specific gene sets associated with the respective macrophage activation programs, we applied weighted gene coexpression network analysis (WGCNA) (Figure 3A), which defines transcriptional modules based on Pearson correlation and determines specific gene-expression patterns for each of the stimulation conditions (Langfelder and Horvath, 2008). We identified 49 distinct coexpression modules containing 27 to 884 genes per module. The expression data from different genes within each calculated module were used to determine the module eigengenes (ME, the first principle component of the respective module), which were correlated to the 29 experimental conditions. As examples, we visualized the expression of the eigengenes of modules 8, 15, and 30 (Figure S3A). The resulting ME-to-condition correlation was then visualized as a heatmap (Figure 3B, summarized in Tables S2A and S2B). Whereas the classical M1 and M2 stimuli showed prominent ME patterns (modules 8 and 15, respectively), other stimuli clearly displayed divergent patterns further supporting a spectrum model of macrophage activation. For instance, stimulation with TNF, PGE_2_, and P3C (TPP, M^TPP^) induced a strong signal in modules 30, 32, and 33, which were not present in IFN-γ or IL-4 stimulated cells. TNF, PGE_2_, and TLR activation have been linked to chronic granulomatous inflammation such as in tuberculosis or granulomatous listeriosis (Marino et al., 1997; Popov et al., 2006; Shay and Celeste Simon, 2012). Genes including *CD25*, *COX-2*, *IL10*, and indoleamine 2,3-dioxygenase (*IDO*) are expressed in macrophages in human granulomatous structures and are induced in human macrophages after stimulation with the combination of the aforementioned factors (TNF, PGE_2_, TLR2 ligand P3C [TPP, M^TTP^]) (Popov et al., 2006; Popov et al., 2008). This suggests that these host factors shape the transcriptional program during chronic inflammation.

As a next step, we used the modules correlated with the specific stimulation conditions IFN-γ, IL-4, and TPP to link respective module genes to biological information. We combined Gene Ontology Enrichment Analysis (GOEA) based on the module genes followed by network visualization of enriched GO-terms using BiNGO and EnrichmentMap (Figure 3C; Figure S3B). This analysis confirmed the major functional differences between IFN-γ and IL-4 differentiated macrophages, e.g., that an M1 response was associated with induction of inflammatory response genes, while these genes were depleted in the M2 response (Figure S3B). More importantly, TPP signaling induced a gene-expression pattern associated with chronic inflammation, including GO terms such as “chronic inflammatory response.” We confirmed our approaches by interrogating these gene sets with the GO analysis, pathway analysis, and transcription factor (TF) binding prediction tools provided by InnateDB, with the same outcome (Tables S2C–S2K). As a next step, we determined TFs within the IFN-γ, IL-4, and TPP-associated modules by using Genomatix and visualized them as correlation networks (Figure 3D). This analysis revealed *STAT1* as a central hub in the IFN-γ-condition and *STAT6* as a hub in the IL-4-condition. We also identified additional TFs that are linked to these activation programs, e.g., *STAT2*, *IRF7*, and *IRF9s* for IFN-γ activation and *IRF4* and the forkhead box proteins *FOXQ1* and *FOXD2*, which were not previously associated with the IL-4 activation network. For macrophages stimulated with TPP, the TF network also included *STAT4*, as well as TFs associated with negative regulation of TLR signaling (*HEY1*; Hu et al., 2008), macrophage activation (*TGIF1*; Ramsey et al., 2008), or TFs at the interface between inflammation and metabolism (*HIF1A*; Shay and Celeste Simon, 2012). Other TFs identified in this network have not yet been linked to macrophage activation as determined by pubatlas.org-based literature mining (data not shown). WGCNA revealed activation-associated gene sets responsible for important biological functions of different macrophage populations. These gene sets harbor specific TF networks including well-established TFs associated with major activation programs but also TFs not previously associated with macrophage activation programs. Taken together, this large data set of macrophage activation forms the basis for the establishment of transcriptional networks that are linked to specific activation signals in human macrophages.

### Distinct Phenotype and Function of Macrophages Activated by TNF, PGE_2_, and TLR2 Ligand

**Figure 4:**
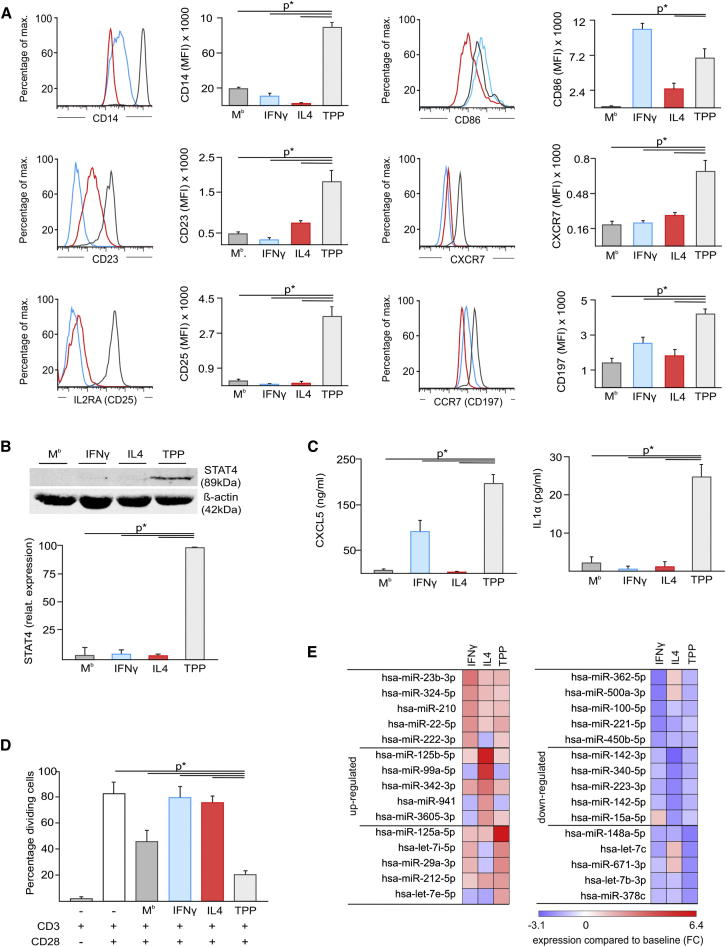
Phenotypic and Functional Characterization of Macrophages Stimulated with TNF, PGE_2_, and P3C (A) Flow cytometry of CD14, CD23, CD25, CD86, CXCR7, CD197, in M^b^ (dark gray), IFN-γ (light blue), IL-4 (red), and TPP (light gray) activated macrophages. Mean fluorescence intensities (MFI) of at least three independent experiments are shown (mean and SEM; p^∗^ < 0.05 Student’s t test). (B) Immunoblot analysis of STAT4 and β-actin. STAT4 expression was normalized to β-actin expression and set to 100% in M^TPP^ (TPP). (C) CXCL5 and IL1α in supernatants of macrophage cultures. (D) T cell activation by CD3 and CD28 beads in presence or absence of macrophages assessed by CFSE dilution. (E) Heatmap showing fold changes of highly abundant miRNAs up- or downregulated (FC > 2, FDR adjusted p value < 0.05) in M1 (IFN-γ) or M2 (IL-4), or M^TPP^ (TPP) compared to M^b^ (baseline). Fold changes colored from blue to red. For Figures 4A–4D, mean ± SEM (p^∗^ < 0.05, Student’s t-test) are shown.

We used the stimulation condition TPP (M^TPP^), which is associated with chronic inflammation, to demonstrate phenotypic and functional differences to macrophages stimulated with IFN-γ or IL-4. As demonstrated by CCM (Figure 1I) and WGCNA (Figure 3), M^TPP^ differed considerably in their genomic signature from M1 or M2 macrophages. By using differentially expressed genes (FC > 2, FDR adjusted p value < 0.05) between M^TPP^ (TPP), M1 (IFN-γ), M2 (IL-4), and M^b^ we identified cell surface markers expressed selectively on M^TPP^. A total of 51 cell surface markers were elevated in M^TPP^ but not M1, M2, or M^b^. By using flow cytometry, we confirmed significantly elevated expression for CD14, CD23, CD25, CXCR7, and CD197 on M^TPP^ (p value < 0.05), while CD86 was elevated on both M1 and M^TPP^ (Figure 4A). We also identified a set of TFs induced in M^TPP^ but not in M1 or M2, among them STAT4. Analysis of STAT4 protein expression clearly confirmed that STAT4 is only induced in M^TPP^ (Figure 4B). Further differences between M^TPP^ and macrophages stimulated with IFN-γ (M1) or IL-4 (M2) were observed for soluble effector molecules where, e.g., CXCL5 secretion was significantly induced by M^TPP^ and to a lesser extent by M1 (p value < 0.05), but not M2, and IL-1α was only secreted by M^TPP^ (Figure 4C). No difference in CD3- and CD28-stimulated T cell proliferation was observed in the presence of M1- or M2-activated macrophages, whereas M^b^ reduced T cell proliferation although not statistically significantly (p value < 0.05) (Figure 4D). However, M^TPP^ showed a strong inhibitory effect, clearly demonstrating that macrophage activation by TPP induced an effector program distinct from M1 and M2 macrophages. Because transcriptional programs are further modulated on posttranscriptional level we assessed the global spectrum of miRNA expression by miRNA-Seq (Figure 4E). Again, M^TPP^ clearly differed from M1 and M2 activation at the miRNA level: M^TPP^ had elevated hsa-miR-125a-5p expression and a lack of M1- (hsa-miR-23b-3p) or M2-associated microRNAs (miRNAs) (e.g., hsa-miR-125b-5p, hsa-miR-99a-5p). Similarly, a set of miRNAs was significantly reduced in M^TPP^ compared to M1 or M2 activation (FDR adjusted p value < 0.05). Therefore, macrophages differing in their global transcriptional program from M1 or M2, such as M^TPP^ macrophages, are also phenotypically and functionally distinct, further supporting the spectrum model of macrophage activation.

### Macrophage Activation Model Can Be Used to Predict Macrophage Programs In Vivo

**Figure 5:**
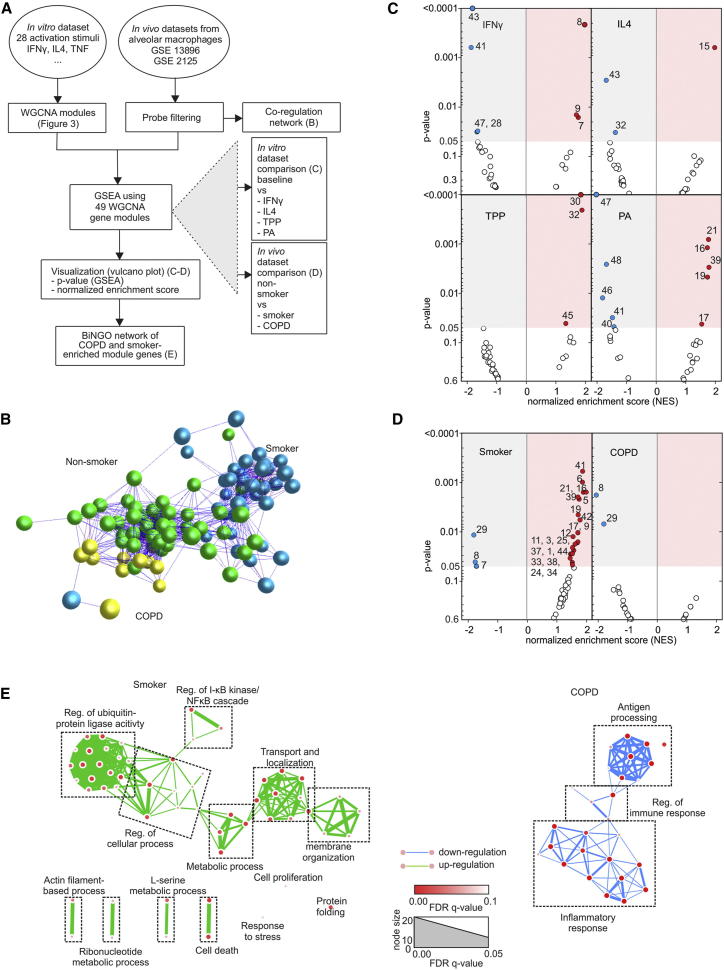
Enrichment of Activation Modules in Human Alveolar Macrophages (A) Schema describing the workflow for Figure 5. (B) Correlation network of human alveolar macrophages (n = 100) from two studies (Shaykhiev et al., 2009; Woodruff et al., 2005) using 374 genes differentially expressed between nonsmokers (n = 39) and smokers (n = 49) or COPD (n = 12) patients (FC > 2.0, FDR adjusted p value < 0.05). (C) Volcano plots of normalized enrichment scores (NES) and enrichment p values based on GSEA using WGCNA modules defined in Figure 3. Shown are data for the stimuli IFN-γ, IL-4, TPP, and palmitic acid (PA). Red circles show gene sets positively significantly enriched (NES > 1, p value < 0.05); blue circles show gene sets significantly depleted (NES < −1, p value < 0.05). (D) Volcano plots of normalized enrichment scores (NES) and enrichment p values of the same gene sets applied to data from alveolar macrophages derived from smokers and COPD patients. Representative results of several permutation runs of GSEA. (E) Network visualization of GOEA of positively enriched modules (p value < 0.01) for smokers (modules 41, 6, 21, 16, 39, 5, 19, and 42) and negatively enriched modules (p value < 0.01) for COPD patients (modules 8, 29) using BiNGO and EnrichmentMap. Red nodes represent enriched GO-terms, node size, and color represent corresponding FDR-adjusted enrichment p values (q values).

To address whether specific activation programs such as those described in Figure 3 can be found in human tissue macrophages, we compiled two data sets of human alveolar macrophages obtained by bronchoalveolar lavage (Shaykhiev et al., 2009; Woodruff et al., 2005) consisting of samples from nonsmokers, smokers, and COPD patients. Following filtering steps, data structure analysis, and data visualization, gene-set enrichment analysis (GSEA) was performed (Figure 5A). Three major clusters reflecting the three patient groups were revealed by CRA (Figure 5B) supporting distinct transcriptional programs in macrophages from the three groups. Next, stimulus-specific gene modules identified by WGCNA (Figure 3) were utilized as 49 gene sets from in vitro conditions for enrichment analysis. As positive controls, we applied GSEA to the comparison of IFN-γ-, IL-4-, TPP-, and palmitic acid (PA)-stimulated macrophages with unstimulated macrophages. We calculated normalized enrichment scores (NES), which were plotted against enrichment p values in a Volcano plot (Figure 5C). As expected, the highest positive NES and lowest p values were observed for those gene sets (gene modules) that were most significantly enriched (NES > 1, p value < 0.05) in the WGCNA analysis for the respective stimuli (IFN-γ, IL-4, TPP, and PA). We then applied GSEA to the patient sample groups (smoker, COPD) in comparison to nonsmokers (Figure 5D). Unexpectedly, in smokers a glucocorticoide (GC)-associated gene module (41, WGCNA) was most significantly enriched (p value < 0.05) followed by several gene modules associated with free fatty acid but also IL-4 and TPP stimulation, suggesting a complex network of stimuli acting on alveolar macrophages in smokers. In contrast to previous literature (Shaykhiev et al., 2009), we did not see an enrichment of IL-4-IL-13-associated signatures in COPD patients with our data-driven approach (Figure 5D). Rather we found a complete loss of the profound signature of enriched modules observed in smokers. Concurrently, the most significantly depleted (NES < −1, p value < 0.05) WGCNA module in COPD patients was module 8 (linked to IFN-γ stimulation), which was also significantly depleted in smokers (Figure 5D). Network visualization of GOEA further supported complex and profound transcriptional changes in alveolar macrophages from smokers while cells from COPD patients were rather characterized by loss of antigen processing, inflammatory response, and regulation of immune response, consistent with a depletion in the IFN-γ linked module (Figure 5E). Applying the WGCNA- and GSEA-defined macrophage differentiation programs to human ex vivo tissue macrophages, we have uncovered a hitherto unexplored biology in alveolar macrophages from smokers and COPD patients.

### Common Denominators of Macrophage Activation

**Figure 6:**
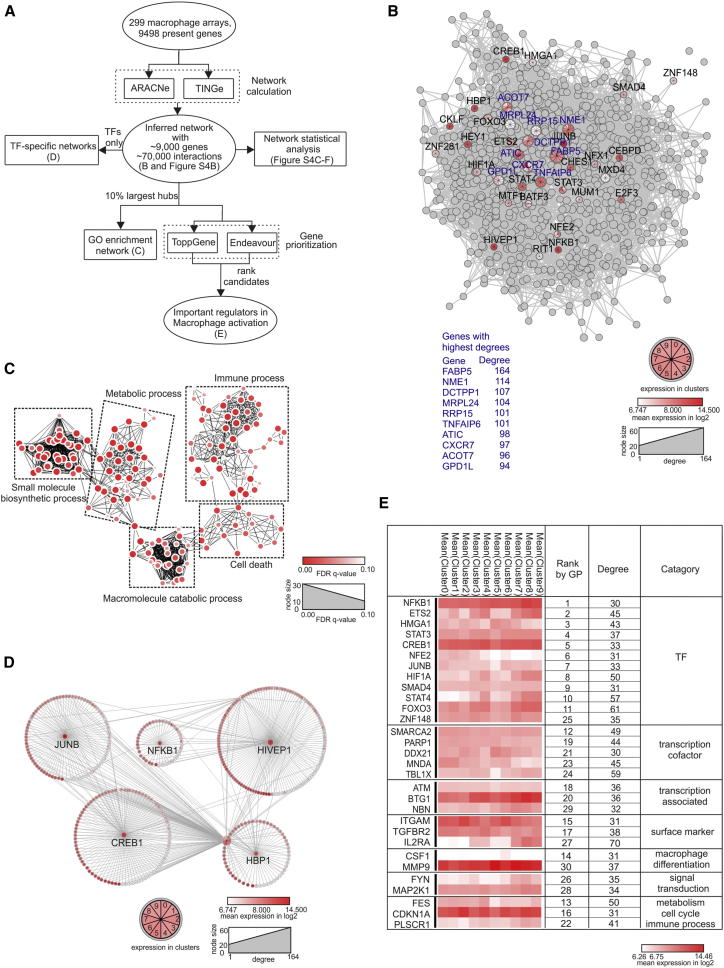
Macrophage Activation Network Calculated by ARACNe (A) Schema describing the workflow for Figure 6. (B) Visualization of the 10% largest hub genes of the ARACNe predicted macrophage regulatory network (n = 299 transcriptomes). For the top ten genes (highest degree of connectivity, blue) and TFs, mean expression values (log2, derived from the ten clusters in Figure 1I) are highlighted in red colors. Node size reflects degree of connectivity. (C) Network visualization of GOEA using BiNGO and EnrichmentMap on hubs shown in (B). Red nodes represent enriched GO-terms, and node size represent FDR-adjusted enrichment p value (q value). Edge thickness represents overlap of genes between neighbor nodes. (D) Subnetworks of the five most highly expressed TFs from all hubs shown in (B). First neighbors are surrounding corresponding TFs. Each gene is multicolored according to its mean expression (log2) in ten clusters (from Figure 1I). (E) Top 30 putative candidates after Gene Prioritization (GP) of 869 hubs. Mean expression (log_2_) from each cluster is displayed as a heatmap. Categorization is according to cellular functions. See also Figures S4 and S5 and Table S3.

While we clearly extended the concept of macrophage polarization (M1 versus M2) to a spectrum model, our large data set also allowed us to define common denominators of macrophage activation. To define these common macrophage activators, we used reverse network engineering (RNE) utilizing ARACNe (Algorithm for the Reconstruction of Accurate Cellular Networks, Figure 6A) (Margolin et al., 2006). We used the 9498 genes present in at least one stimulation condition (Table S1D) to generate a so-called all-versus-all network (Bonferroni corrected p value 10^−7^) by predicting interactions based on mutual information between each gene pair computed from the expression profiles (Figure S4A; Table S3A; for further technical details see Supplemental Information). We identified 66,744 interactions resulting in an average degree of connectivity of 14.7, meaning one gene is involved in 15 transcriptional interactions on average (Figure 6B; Table S3B; Figure S4B). We confirmed these findings with a second RNE approach (TINGe, Tool for Inferring Network of Genes) (Aluru et al., 2013), which demonstrated high similarity in the number of interactions, the average degree of connectivity and the rank of hubs based on degrees of connectivity as determined by ARACNe (Table S3C; Figure S4C). We summarized the network statistical properties (Figures S4D–S4F), since the entire network is too complex to be displayed. The top 10% of hub genes inferred in the network (n = 869 most interconnected genes) collectively participated in 30,431 interactions (Figure 6B). In the ten most highly interconnected genes, we identified *FABP5*, which has recently been implicated in lipid metabolism and inflammation crosstalk (Furuhashi et al., 2011), and *TNFAIP6*, a negative feedback regulator of myeloid cell activation (Choi et al., 2011). However, according to a pubatlas.org search (Table S3D), little is known about the role of the other most highly interconnected genes during macrophage activation, suggesting that RNE approaches reveal unknown aspects of macrophage activation. To further understand the biological processes of the top 10% of hub genes, we performed GOEA with visualization of GO enrichment networks by using BiNGO (Figure 6C; Table S3E). This GO-term network subdivided into five major clusters, one of which was related to immune response processes (especially terms associated with “regulation of activation”). However, other clusters were associated with cell death, biosynthetic processes of small molecules, and metabolic and catabolic processes, which also constitute major but underappreciated aspects of macrophage activation.

By using the TFCat database (Fulton et al., 2009), we identified 27 TFs in the top 10% hub genes (TFs, Figure 6B). We reasoned that the most highly expressed TFs are the most relevant for macrophage activation and therefore ranked them by average expression and generated a network of the top five TFs (*JUNB*, *NFKB1*, *HIVEP1*, *CREB1*, and *HBP1*) (Figure 6D; Table S3F). Roles in macrophage activation have been established for all of these TFs, except for the zinc finger protein HIVEP1: JUNB (part of the AP1 complex), NF-κB (global activator), and CREB1 (inducing survival signals) (Wen et al., 2010). HBP1 has been linked to differentiation of malignant myeloid cells and to the regulation of other important TFs including PU.1, RUNX1, JUNB, or CEBP (Yao et al., 2005). By using position-weight matrices, we predicted binding of the 27 TFs to the gene loci of the top 10% hub genes (Table S3G). Twenty-six out of 27 of these TFs showed significantly enriched binding prediction (p value < 0.05).

As a complementary approach, we also applied two gene prioritization tools, ToppGene (Chen et al., 2009) and Endeavour (Tranchevent et al., 2008) to rank the potential association and closeness of the top 10% hub genes with macrophage cellular programs using the macrophage lineage TFs *RUNX1* and *SPI1* (PU.1) as test genes. Of note, the top 11 ranked genes were TFs and in total, 20 of the 30 top ranked genes are associated with transcriptional regulation (Figure 6E). In addition to *NFKB1*, *JUNB*, and *CREB1*, we identified additional TFs already associated with macrophage activation (*STAT3*), as well as other TFs not yet associated with macrophage activation (*HMGA1*, *NFE2*, *ZNF148*, *SMARCA2*, *DDX21*, *MNDA*, *TBLX1*). Several macrophage-activation markers (e.g., *MMP9* and *CSF1* [M-CSF]) were also strongly linked to macrophage activation in this analysis. Furthermore, a strong enrichment of PU.1 binding and permissive histone marks H3K4me3 at the loci of the 869 major hub genes indicate that these genes are likely to be highly transcribed during macrophage activation (Figure S5; Supplemental Information). Together, this RNE analysis identified five distinct clusters of biological processes as part of the macrophage activation process, confirmed the involvement of known transcriptional regulators such as NFKB1, and identified unexpected yet unexplored candidate regulators.

### Refinement of Core Genes of Murine Tissue Macrophages using Human Macrophage Activation Signatures

**Figure 7:**
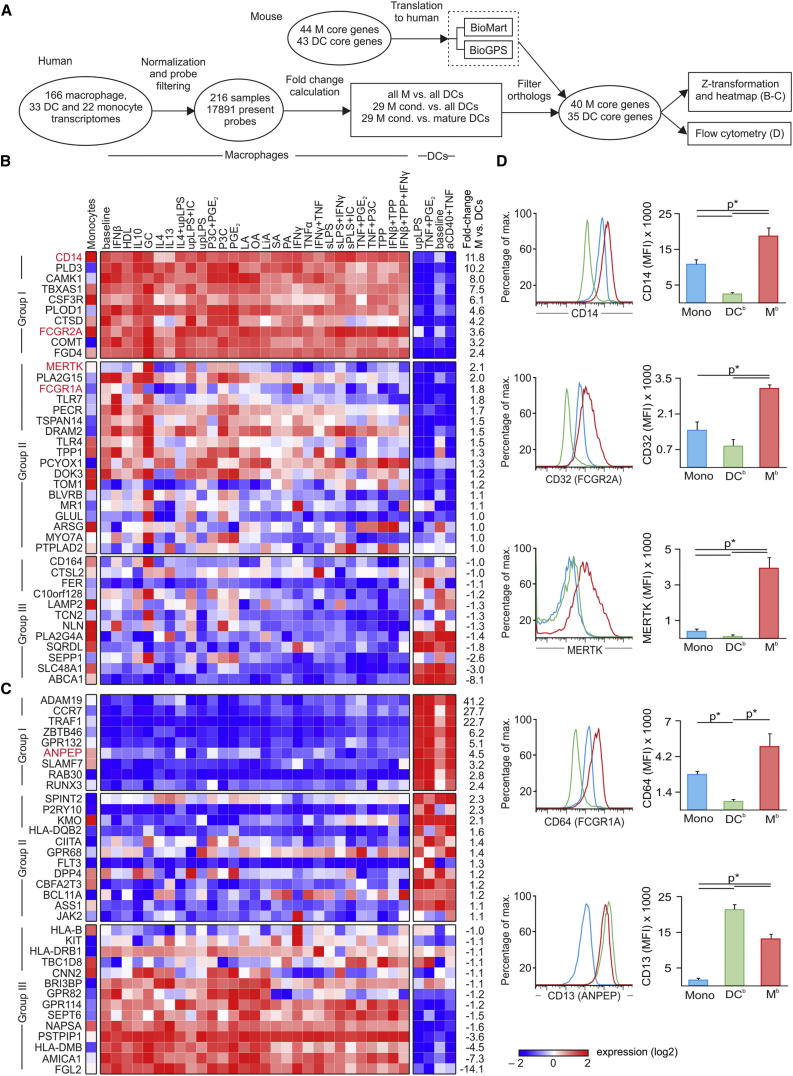
Expression of murine macrophage and dendritic cell signature genes in humans (A) Schema describing the workflow for Figure 7. M, macrophage. (B and C) Heatmap (standardized and scaled log_2_ expression) of human orthologs of murine (B) macrophage signature genes (Gautier et al., 2012) and (C) DCs signature genes (Miller et al., 2012) in monocytes, 29 macrophage stimulation conditions, and monocyte-derived DCs (baseline = GM-CSF+IL4). TPP = TNF+PGE_2_+P3C. (D) Flow cytometric analysis for surface markers CD14, CD32, MERTK, CD64, CD13 on human monocytes (Mono, blue), baseline DCs (DC^b^, green), and baseline macrophages (M^b^, red). Mean fluorescence intensities (MFI) of at least three independent experiments (mean and SEM; p^∗^ < 0.05 Student’s t test). See also Table S4.

Comparative transcriptomics of immune cells between human and mouse provides a framework for the use of model systems in the context of human biology and disease (Shay et al., 2013). We therefore propose a general strategy for how our resource of human macrophage transcriptomes can be linked to murine data (Figure 7A). First, we visualized the expression of the human orthologs of the ImmGen defined core macrophage (Figure 7B) and DC (Figure 7C) genes in human macrophages and monocyte-derived DCs under the different stimulation conditions. We visualized the expression values as heatmaps of genes ranked by overall differential expression between human macrophages and DCs (Figures 7A–7C; Table S4). Within the core signature genes defining murine macrophages, we identified three groups of genes (Figure 7B). The first group of genes (1) had high conservation of differential expression between human macrophages and DCs irrespective of macrophage activation, whereas the second group of genes (2) was characterized by loss of differential expression after activation with certain stimuli. The third group of genes (3) was either not differentially expressed between human macrophages and DCs or showed even opposite regulation (Figure 7B). A similar grouping was found for the core signature genes of DCs (Figure 7C). This approach identified cell surface markers (*CD14*, *FCGR2A* [CD32], *MERTK*, *FCGR1A* [CD64], *CD13* [ANPEP]) that distinguish human macrophages from both DCs and CD14^+^ blood monocytes by flow cytometric analysis (Figure 7D). We propose that this set of cell surface markers should be used for the discrimination between macrophages and DCs in both species. Overall, this comparative transcriptomics approach has refined the core signatures for macrophages and DCs determined in mice to make them also applicable to human macrophages. This will further improve the interpretation of data obtained in species other than human and will guide animal model design to better reflect relevant human biology.

## Discussion

The generation of this large and unique transcriptomic data set of human macrophage activation represents an important step forward in understanding how macrophages integrate and compute signals from their local microenvironment under inflammatory conditions. The extension of macrophage activation from M1 versus M2 polarization to a spectrum model opens new avenues to study macrophage activation in the context of human diseases. For example, network-based description of global but also input signal-specific transcriptional programming could form the basis for further studies linking defined activation programs with in vivo human macrophage biology. In fact, the lack of any major inflammatory signals in human alveolar macrophages derived from COPD patients was an unexpected—but from a clinical perspective, highly important—result. This result might actually reflect clinical observations demonstrating inefficiency of anti-inflammatory treatment regimens in COPD patients making it necessary to search for alternative strategies (Barnes, 2013). A better understanding of the transcriptional regulation of human macrophages could help to selectively target specific macrophage subsets therapeutically and thereby could spare other cell types.

Integrating human and murine transcriptome data sets will aid to prioritize and focus future work in animal models. In this respect, overlaying our human data onto the ImmGen-derived core signatures of DCs and macrophages classified several genes to be conserved in expression regulation, whereas others clearly are not conserved. In the future, these studies have to be extended comparing macrophages from the same tissues in both species in homeostasis and pathophysiology. Alternatively, the identification of human-specific regulation of gene expression will require new methodologies to study gene regulation in an entirely human context, and without the respective animal models.

Beyond aspects comparing murine and human macrophages, this resource data set can help to answer open questions concerning differential activation of human macrophages with closely related stimuli, e.g., ultrapure and standard LPS, which have been often used synonymously in prior studies. Similarly, while free fatty acids seem to induce closely related transcriptional programs when compared to the remaining stimulation conditions, we have clear evidence that saturated and unsaturated fatty acids induce distinct transcriptional responses in human macrophages (S.V.S., data not shown).

We anticipate this data set of human macrophage activation to serve as a starting point for future studies into human macrophage biology. In addition to expanding our understanding of human macrophage biology, this resource will contribute to a better understanding of general mechanisms of transcriptional control, as well as the development of new mathematical models for signal integration and new therapeutic strategies in human disease.

## Experimental Procedures

Detailed description of all experimental procedures and links to analytical tools and databases used are provided in Supplemental Information. Abbreviations and description of bioinformatics tools are summarized in Table S5.

### Isolation, In Vitro Culture, and Functional Assessment of Cells under Study

Buffy coats from healthy donors were obtained according to protocols accepted by the institutional review board at the University of Bonn (local ethics votes no. 288/13). Human monocytes, B cells, T cells, and NK cells (Table S1) were purified from peripheral blood mononuclear cells by MACS in accordance with the manufacturer’s instructions. Macrophages (M^b^, baseline) were generated from monocytes by stimulation with GM-CSF or M-CSF for 72 hr and further activated with 28 stimulation conditions (Table S1). DCs were generated by GM-CSF in presence of IL-4 for 72 hr followed by further stimulation with uLPS, TNF+PGE_2_, or αCD40 mAbs+TNF. Cells were phenotypically assessed by flow cytometry using cell lineage and activation markers. Expression of STAT4 was measured by immunoblotting. CXCL5 and IL-1α in cell culture supernatants were assessed by ELISA following the manufacturer’s instructions. The influence of macrophages on T cell activation was measured in an allogeneic mixed lymphocyte reaction where CD3^+^ T cells were stimulated with CD3+CD28 mAbs in presence or absence of differentially activated macrophages. T cell proliferation was assessed 72 hr later using the carboxyfluorescein succinimidyl ester dilution method.

### RNA Isolation, Gene Expression Profiling, and Basic Bioinformatical Analysis

Biotin-labeled cRNA was generated with the TargetAmp Nano-g Biotin-aRNA Labeling Kit for the Illumina System (Epicenter) and then hybridized to Human HT-12V3 and Human WG-6V3 Beadchips (Illumina) and scanned on an Illumina iScan or HiScanSQ system. Exported from Genome Studio (Illumina), all array data (n = 384) were imported into Partek Genomics Suite (PGS) prior to quantile normalization. For all analyses with macrophages only (n = 299), background signals were calculated resulting in 9,498 unique genes to be present in at least one macrophage stimulation condition. miRNA- and chromatin immunoprecipitation sequencing (ChIP-seq) procedures are described in detail in Supplemental Information.

### Coregulation Networks and Comparative Bioinformatics

To describe the structure within the data set, we primarily performed coregulation analysis (CRA) based on Pearson’s correlation coefficients by using BioLayout Express^3D^ (Theocharidis et al., 2009) (Figures 1B–G; Table S1B). We determined ANOVA model-defined variable or differentially expressed genes. To corroborate a stimulus-specific structure in the data set (Figure 1H) or to determine genes that are elevated only in one of the tested conditions (Figure 2), we determined self-organizing maps (SOM) and visualized them after hierarchical clustering (HC) (SOM-clustering). On the basis of the 1,000 most variable genes within the data set, we calculated Pearson’s correlation coefficient for all stimulation conditions and visualized the resulting correlation coefficient matrices (CCM) after HC as a heatmap (Figure 1I). The spectrum model of macrophage activation was established by grouping the samples according to the clusters obtained by the CCM analysis, utilizing the 3D coordinates of the individual macrophage samples determined by CRA, calculating mean vectors for the clusters and plotting the information in a 3D graph using the coordinates of the baseline macrophages (M^b^) as the origin (Figure 1J).

### Biological Processes Based on Weighted Gene Coexpression Network Analysis

To determine biological processes either enriched or depleted in the 28 macrophage stimulation conditions, we first dissected the data set (n = 160 macrophage samples, 72 hr time point) into 49 gene modules by applying WGCNA (Langfelder and Horvath, 2008) (Table S1B; Table S2). The module eigengene (ME) corresponding to the first principal component was calculated for each module and a ME-to-condition correlation visualized as heatmap (Figure 3). For the stimulation conditions IFN-γ, IL-4, and TPP (TNF+PGE_2_+P3C), the gene modules with highest respective lowest correlation scores were used to generate and visualize networks based on GO-enrichment analysis (GOEA) by using BiNGO, EnrichmentMap, and Word Clouding in Cytoscape. Visualization of coregulation of TFs of the same modules was performed by BioLayout Express^3D^.

### Combining WGCNA and Gene Set Enrichment Analysis

To utilize the information from the macrophage activation data set to assess in vivo biology of macrophages, we developed an approach utilizing the WGCNA-based gene modules as gene sets for gene-set enrichment analysis (GSEA, see schema in Figure 5). Two data sets of human alveolar macrophages (GSE13896 [Shaykhiev et al., 2009] and GSE2125 [Woodruff et al., 2005]) comprising samples from 39 nonsmokers, 49 smokers, and 12 COPD patients were combined. GSEA was performed on 49 WGCNA modules (Figure 3) in 10,000 permutations by using PGS. Normalized enrichment scores (NES) together with p values of GSEA were plotted by Volcano plots comparing the stimulation conditions IFN-γ, IL-4, TPP, and PA with M^b^ (as positive control analyses of the overall approach). Similarly, the comparison of nonsmokers with smokers or COPD patients was visualized by Volcano plots. Enriched modules (p value < 0.01) were selected to perform GOEA.

### Reverse Engineering of the Core Macrophage Activation Network

To determine the central hubs of all stimulation conditions reflecting the core macrophage-activation network, we applied two information-theoretic methods, ARACNe and TINGe. Networks were visualized in a force-directed layout in Cytoscape, followed with statistical analysis utilizing the plug-in Network Analysis (Cline et al., 2007). With the cytoscape plug-in MultiColoredNodes (Warsow et al., 2010) mean expression values of the most highly interconnected genes and TFs were visualized.

### Linking Human Macrophage Activation to ImmGen Core Signatures

Human macrophage samples (n = 166), DCs (n = 33), and monocytes (n = 22) were compiled and expression values of the human orthologs of the previously described murine macrophage (Gautier et al., 2012), and DC (Miller et al., 2012) core signatures were plotted as a heatmap of standardized and scaled log_2_ values (Figure 7; Table S4).

## Accession Numbers

Macrophage microarray data, ChIP-Seq data, miRNA-Seq data, and alveolar macrophage microarray data are accessible via super series GSE47189. Preprocessed data are also available at an online resource portal (www.macrophages.uni-bonn.de).

## Supplemental Information

Document S1. Figures S1–S5, Tables S1–S5, and Supplemental Experimental Procedures

Table S1. Gene Expression Microarray Data Sample Information, Related to Figure 1

Table S2. 49 Modules Identified from WGCNA, Related to Figure 3

Table S3. Reverse Engineering of Regulatory Networks by ARACNe and TINGe, Related to Figure 6

Table S4. Comparison of Murine Macrophage and Dendritic Cell Signature Genes Expression across Human Data Set, Related to Figure 7

Table S5. Abbreviations and Descriptions of Used Algorithms and Software, Related to Experimental Procedures

Movie S1. Classical M1 versus M2 Polarization Rotation of coregulation networks with 3, 17, and 29 in vitro conditions in 3-dimensional space (related to Figures 1B, 1E, and 1F).
