# Supplementary material for: Transcriptome-Based Network Analysis Reveals a Spectrum Model of Human Macrophage Activation
Source: Immunity. 2014 Feb 20;40(2):274–88. doi: 10.1016/j.immuni.2014.01.006 (PMC3991396; doi:10.1016/j.immuni.2014.01.006)
Supplement: Document S1. Figures S1–S5, Tables S1–S5, and Supplemental Experimental Procedures [file mmc1.pdf]

Immunity, Volume *40*

Supplemental Information

Transcriptome-Based Network Analysis Reveals a Spectrum Model of Human Macrophage Activation

Jia Xue, Susanne V. Schmidt, Jil Sander, Astrid Draffehn, Wolfgang Krebs, Inga Quester, Dominic De Nardo, Trupti D. Gohel, Martina Emde, Lisa Schmidleithner, Hariharasudan Ganesan, Andrea Nino-Castro, Michael R. Mallmann, Larisa Labzin, Heidi Theis, Michael Kraut, Marc Beyer, Eicke Latz, Tom C. Freeman, Thomas Ulas, and Joachim L. Schultze

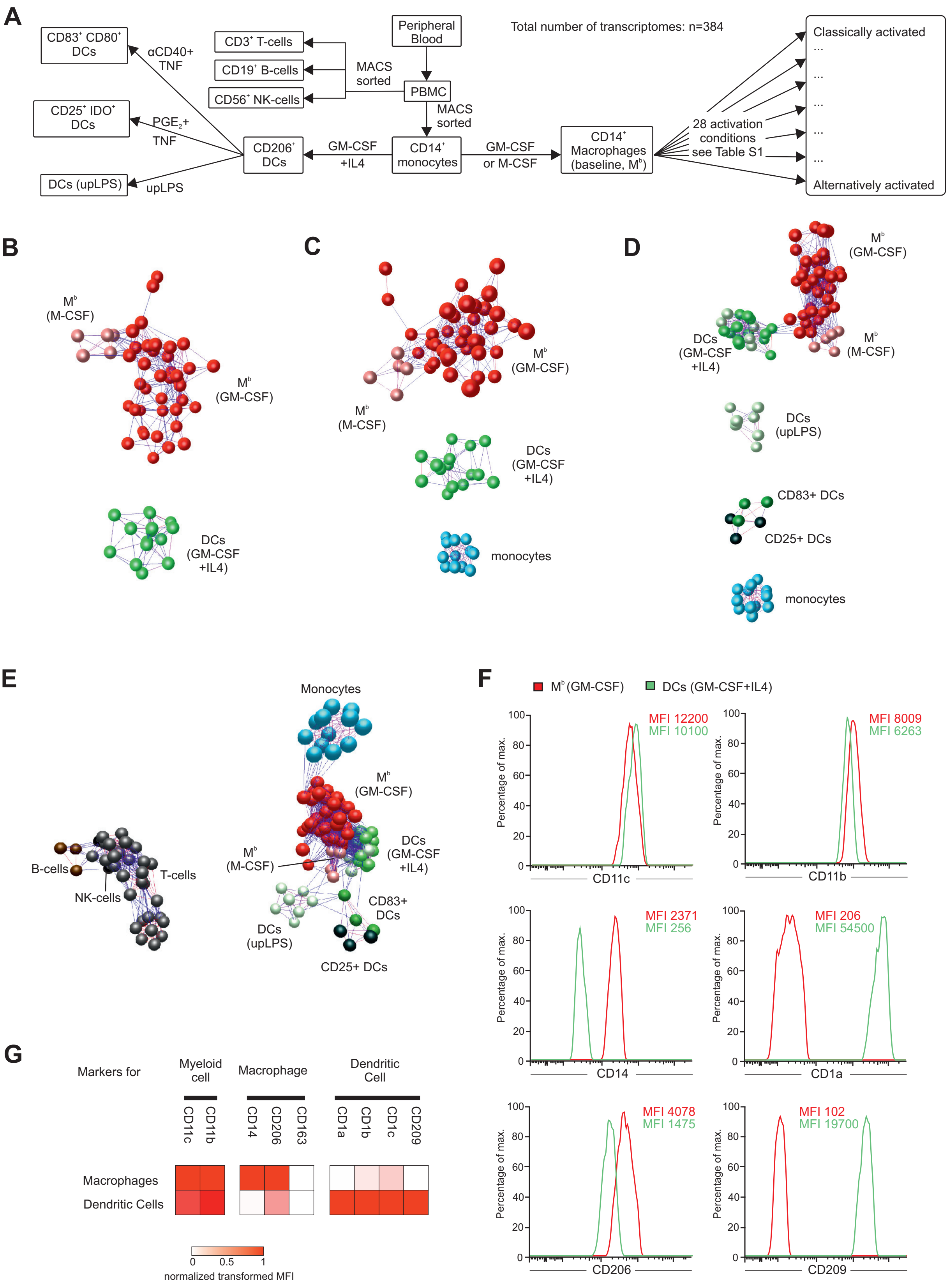

A
Peripheral Blood
PBMC
MACS sorted
CD3+ T-cells
CD19+ B-cells
CD56+ NK-cells
CD14+ monocytes
GM-CSF +IL4
CD206+ DCs
αCD40+ TNF
CD83+ CD80+ DCs
PGE2+ TNF
CD25+ IDO+ DCs
upLPS
DCs (upLPS)
GM-CSF or M-CSF
CD14+ Macrophages (baseline, Mb)
Total number of transcriptomes: n=384
28 activation conditions see Table S1
Classically activated
Alternatively activated
B
Mb (M-CSF)
Mb (GM-CSF)
DCs (GM-CSF +IL4)
C
Mb (GM-CSF)
Mb (M-CSF)
DCs (GM-CSF +IL4)
monocytes
D
Mb (GM-CSF)
DCs (GM-CSF +IL4)
Mb (M-CSF)
DCs (upLPS)
CD83+ DCs
CD25+ DCs
monocytes
E
Monocytes
Mb (GM-CSF)
Mb (M-CSF)
DCs (GM-CSF +IL4)
CD83+ DCs
DCs (upLPS)
CD25+ DCs
B-cells
T-cells
NK-cells
F
Mb (GM-CSF)
DCs (GM-CSF+IL4)
Percentage of max.
CD11c MFI 12200 MFI 10100
CD11b MFI 8009 MFI 6263
CD14 MFI 2371 MFI 256
CD1a MFI 206 MFI 54500
CD206 MFI 4078 MFI 1475
CD209 MFI 102 MFI 19700
G
Markers for
Myeloid cell
Macrophage
Dendritic Cell
CD11c CD11b CD14 CD206 CD163 CD1a CD1b CD1c CD209
Macrophages
Dendritic Cells
0 0.5 1
normalized transformed MFI

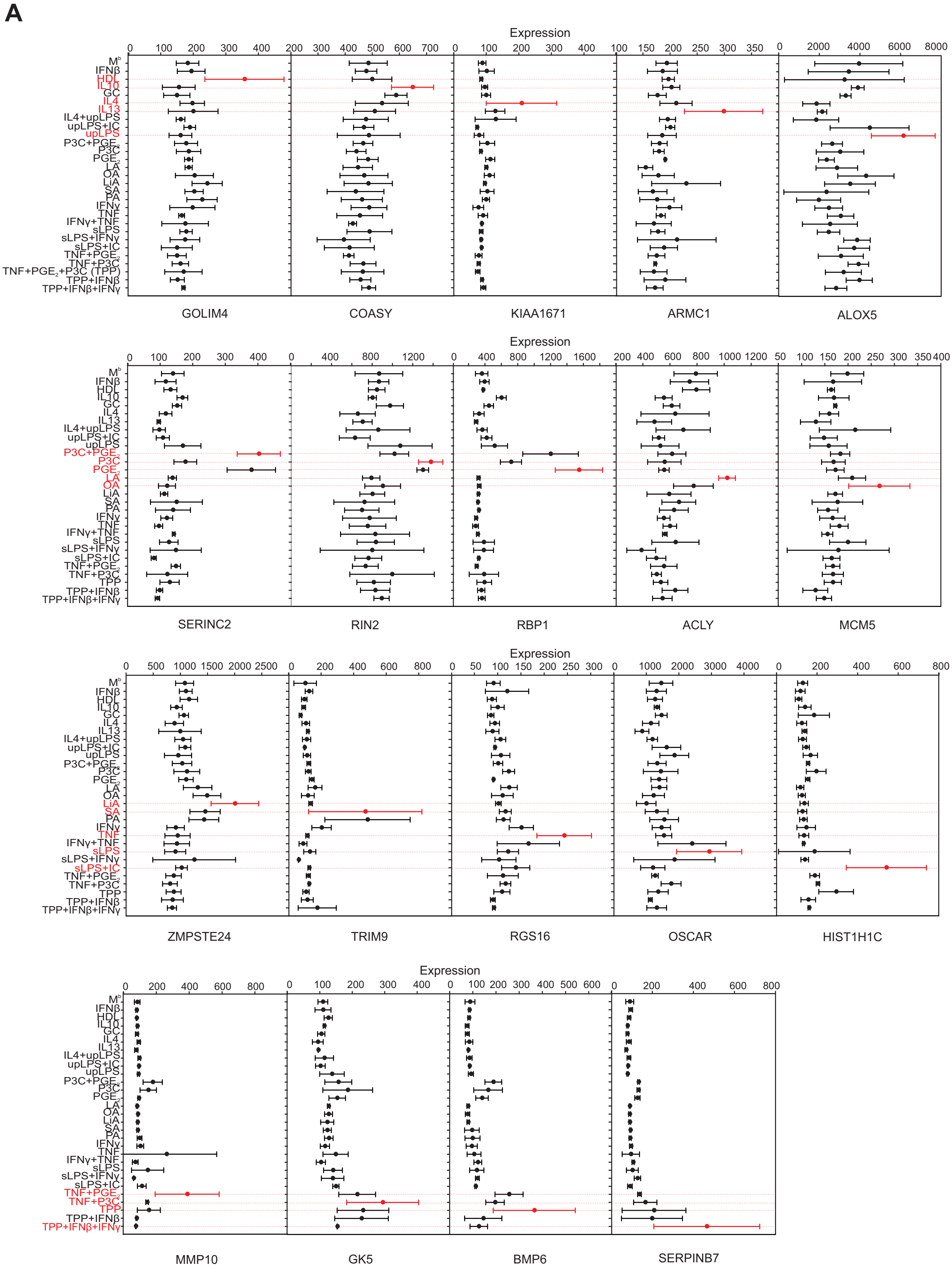
A
Expression
Mᵇ
IFNβ
HDL
IL10
GC
IL4
IL13
IL4+upLPS
upLPS+IC
upLPS
P3C+PGE₂
P3C
PGE₂
LA
OA
LiA
SA
PA
IFNγ
TNF
IFNγ+TNF
sLPS
sLPS+IFNγ
sLPS+IC
TNF+PGE₂
TNF+P3C
TNF+PGE₂+P3C (TPP)
TPP
TPP+IFNβ
TPP+IFNβ+IFNγ
GOLIM4
COASY
KIAA1671
ARMC1
ALOX5
SERINC2
RIN2
RBP1
ACLY
MCM5
ZMPSTE24
TRIM9
RGS16
OSCAR
HIST1H1C
MMP10
GK5
BMP6
SERPINB7

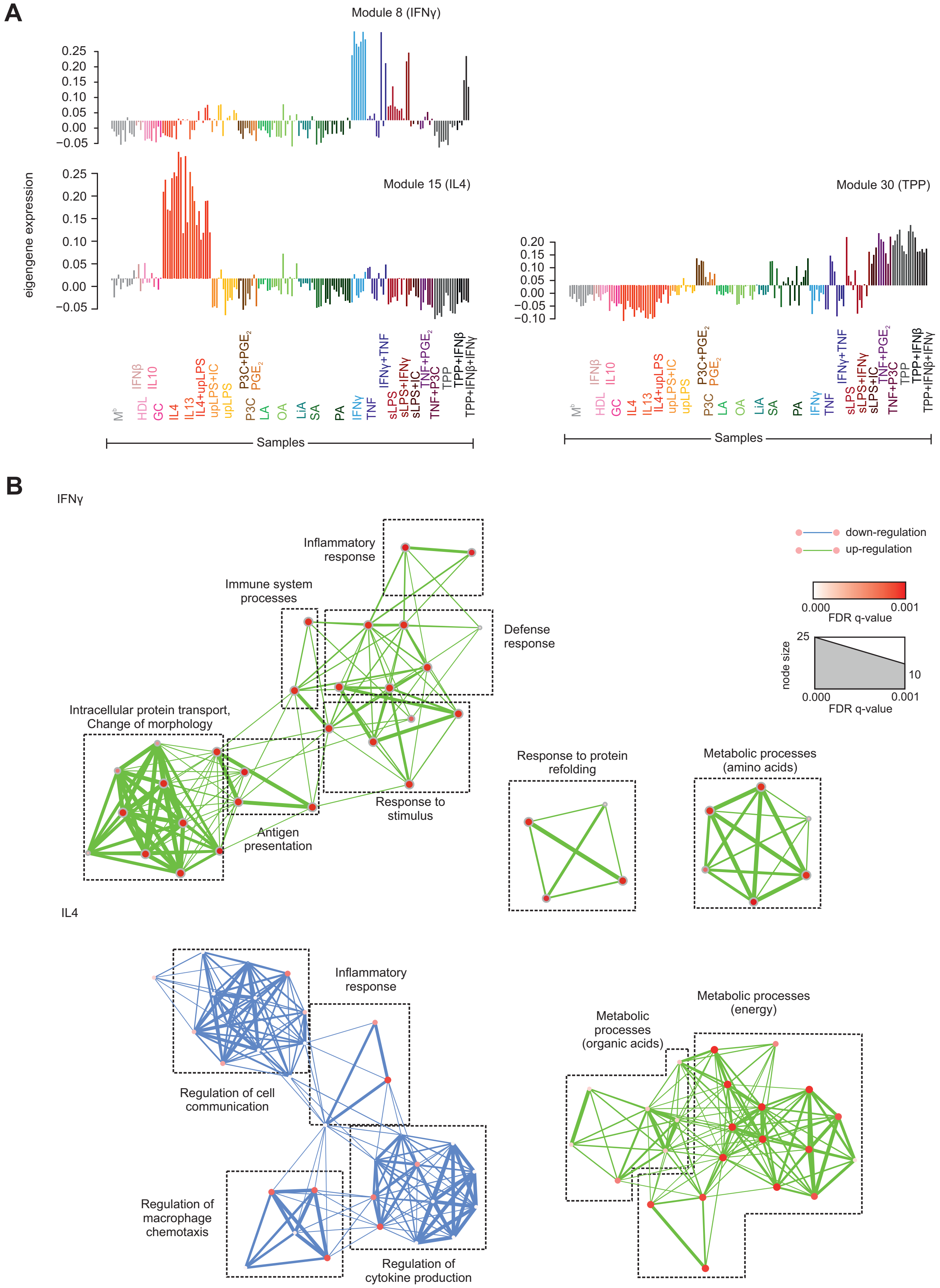

A
Module 8 (IFNγ)
Module 15 (IL4)
Module 30 (TPP)
eigengene expression
Mᵇ
HDL
IFNβ
GC
IL10
IL4
IL13
IL4+upLPS
upLPS+IC
upLPS
P3C
P3C+PGE₂
PGE₂
LA
OA
LiA
SA
PA
IFNγ
TNF
IFNγ+TNF
sLPS
sLPS+IFNγ
sLPS+IC
TNF+PGE₂
TNF+P3C
TPP
TPP+IFNβ
TPP+IFNβ+IFNγ
Samples
B
IFNγ
Inflammatory response
Immune system processes
Defense response
Intracellular protein transport, Change of morphology
Antigen presentation
Response to stimulus
Response to protein refolding
Metabolic processes (amino acids)
down-regulation
up-regulation
FDR q-value
node size
IL4
Inflammatory response
Regulation of cell communication
Regulation of macrophage chemotaxis
Regulation of cytokine production
Metabolic processes (energy)
Metabolic processes (organic acids)

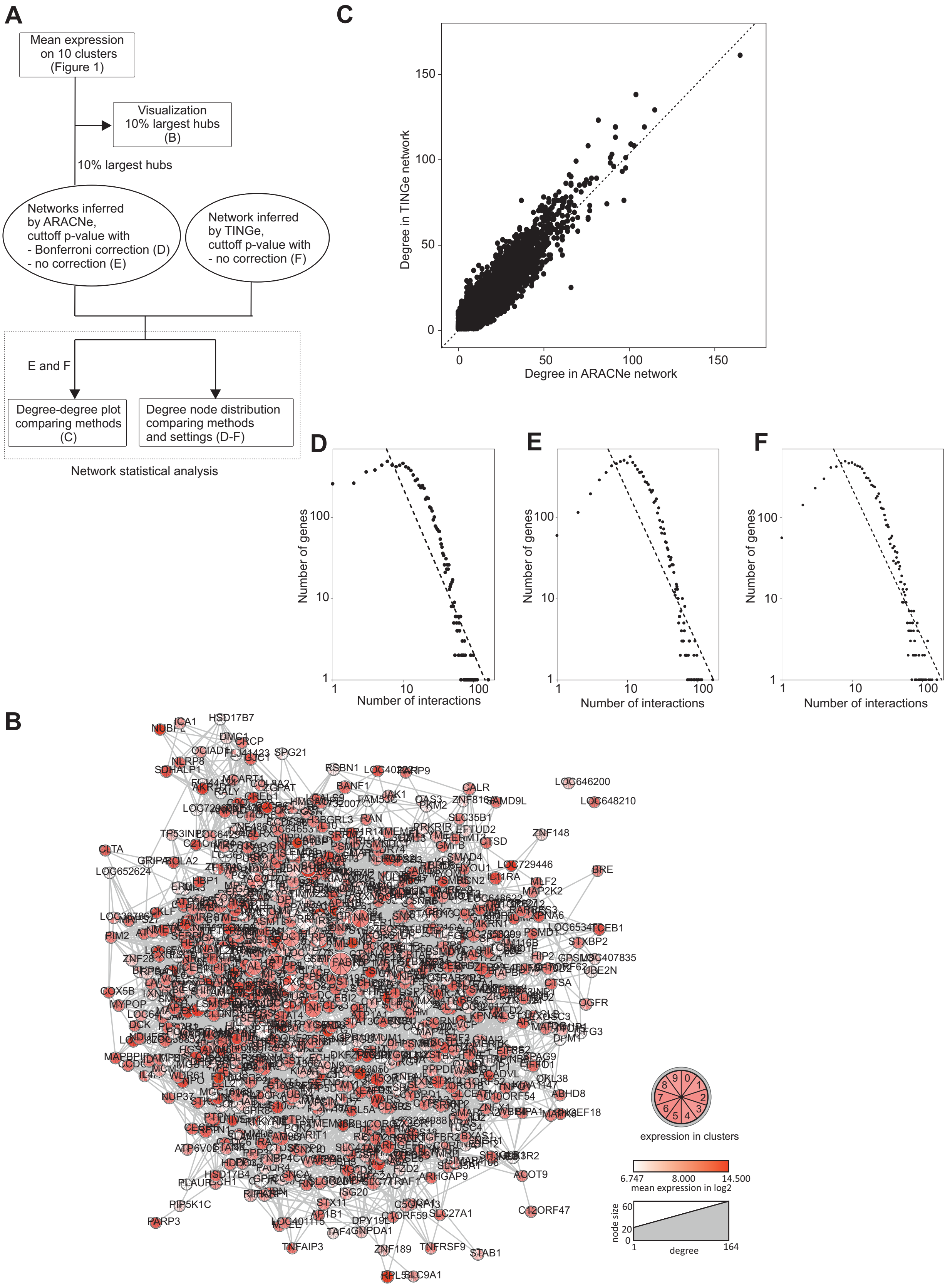
A
Mean expression on 10 clusters (Figure 1)
Visualization 10% largest hubs (B)
10% largest hubs
Networks inferred by ARACNe, cuttoff p-value with
- Bonferroni correction (D)
- no correction (E)
Network inferred by TINGe, cuttoff p-value with
- no correction (F)
E and F
Degree-degree plot comparing methods (C)
Degree node distribution comparing methods and settings (D-F)
Network statistical analysis
C
Degree in TINGe network
Degree in ARACNe network
0
50
100
150
D
E
F
Number of genes
Number of interactions
1
10
100
B
expression in clusters
6.747
8.000
14.500
mean expression in log2
node size
60
20
0
1
164
degree

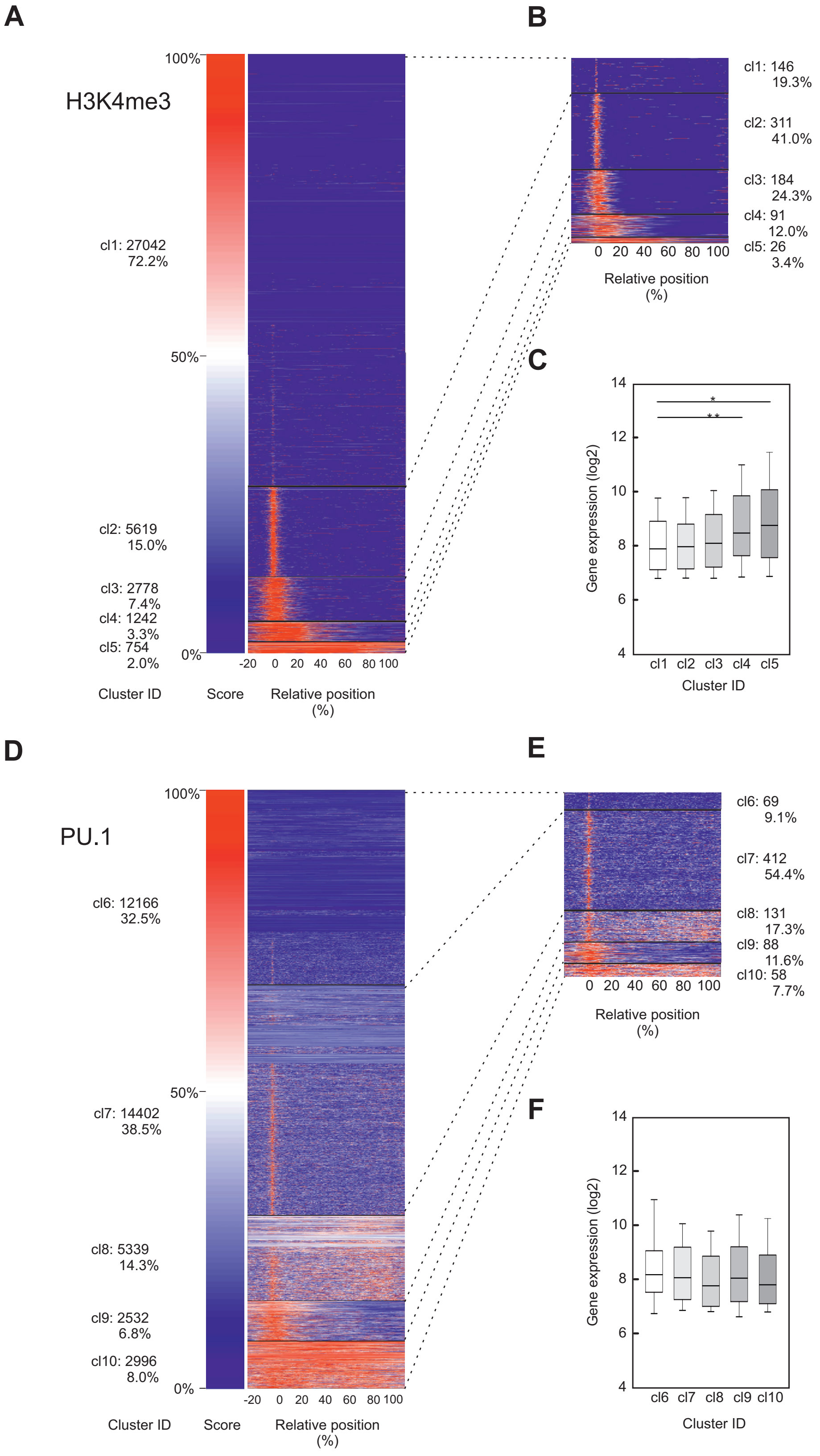

A
H3K4me3
100%
50%
0%
cl1: 27042
72.2%
cl2: 5619
15.0%
cl3: 2778
7.4%
cl4: 1242
3.3%
cl5: 754
2.0%
-20 0 20 40 60 80 100
Cluster ID
Score
Relative position (%)
B
cl1: 146
19.3%
cl2: 311
41.0%
cl3: 184
24.3%
cl4: 91
12.0%
cl5: 26
3.4%
0 20 40 60 80 100
Relative position (%)
C
14 12 10 8 6 4
Gene expression (log2)
*
**
cl1 cl2 cl3 cl4 cl5
Cluster ID
D
PU.1
100%
50%
0%
cl6: 12166
32.5%
cl7: 14402
38.5%
cl8: 5339
14.3%
cl9: 2532
6.8%
cl10: 2996
8.0%
-20 0 20 40 60 80 100
Cluster ID
Score
Relative position (%)
E
cl6: 69
9.1%
cl7: 412
54.4%
cl8: 131
17.3%
cl9: 88
11.6%
cl10: 58
7.7%
0 20 40 60 80 100
Relative position (%)
F
14 12 10 8 6 4
Gene expression (log2)
cl6 cl7 cl8 cl9 cl10
Cluster ID

SUPPLEMENTAL INFORMATION

Figure Legends to Figure S1-S5

Figure S1. Relationship of monocyte-derived macrophages (M^b) with other cell types (related to Figure 1). **A** Schema of isolation and generation of cells used in this dataset. **B** Sample correlation network (visualized in 3-dimensional space) of monocyte-derived macrophages (baseline, M^b) induced by M-CSF or GM-CSF with monocyte-derived cells (DCs) induced by GM-CSF+IL4. Sample correlation networks additionally including **C** monocytes, **D** $CD83^+$ DCs, $CD25^+$ DCs and upLPS-stimulated DCs, **E** T-cells, B-cells and NK-cells. **F** Representative histograms of expression of cell surface molecules CD11c, CD11b, CD14, CD1a, CD206, CD209 on M^b (GM-CSF) and DCs (GM-CSF+IL4). **G** Heatmap of normalized transformed mean fluorescence intensities (MFI) of at least 25 independent experiments of the markers CD11c, CD11b, CD14, CD206, CD1a, CD1b, CD1c, and CD209.

Figure S2. Genes with selective expression associated with distinct stimuli (related to Figure 2). A Absolute expression counts (mean ± SD) of genes defined by SOM clustering to be highly expressed for a particular stimulation condition. Shown here are genes enriched in those conditions, not shown in **Figure 2**.

Figure S3. Activation-specific genes revealed by Weighted Correlation Network Analysis (related to Figure 3). **A** Visualization of the eigengene expression of modules 8, 15, and 30 in the 29 stimulation conditions. **B** Network visualization of Gene Ontology Enrichment Analysis (GOEA) of modules 7-9 (positively correlated) and 43, 44, 48 (negatively correlated) for IFNγ stimulation, 13-15 (positively correlated) and 30, 5, 32 (negatively correlated) for IL4 stimulation using BiNGO and EnrichmentMap. Red nodes represent enriched GO-terms, node size corresponding enrichment p-value. Edge thickness shows overlap of genes between neighbor nodes.

Figure S4. Comparison of two reverse engineering approaches (related to Figure 6). **A** General workflow of network calculation using ARACNe or TINGe. Three parameter settings were performed for the comparison, and subsequently, the inferred networks were compared by network statistical analysis. **B** ARACNe predicted macrophage network with Bonferroni correction. 869 largest hubs are shown as in **Figure 6B**. Each gene is multi-colored according to its mean expression (log2) in 10 clusters. Starting from background value 6.747 to 14.500, the color is changing from white to red. Node size is proportional to degree of connectivity. **C** Comparison of topology of two networks from two algorithms: ARACNe and TINGe. Shown is the degree of connectivity of 9485 genes within the networks. Parameters used for network generation are identical (**Table S3A, C**). **D-F** Degree node distribution of the three major networks generated from different tools or settings: **D** ARACNe cutoff p-value with Bonferroni correction, **E** ARACNe without Bonferroni correction, and **F** TINGe without p-value adjustment. In each plot, the number of genes with the same number of interactions (from 1 to 164) fits to a power law (dash line) in logarithmic range. This indicates that they are scale-free networks.

Figure S5. Permissive histone marks H3K4me3 and PU.1 binding sites at major hub gene loci (related to Figure 6). ChIP-seq for H3K4me3 (**A**-**C**) and PU.1 (**D**-**F**) were performed on different macrophage populations. Using all Ensembl genes as bait, k-means clustering was performed. Genes were first ranked by cluster and within each of the 5 clusters by signal intensity at the transcription start site (TSS). Enriched ChIP-seq signals were depicted in red and signal location is displayed using normalized gene loci relative to the TSS (in percent). **A** Concatenated data for H3K4me3 from M^b, IL4-, IFNγ- or TNF+PGE_2+P3C (TPP) stimulated macrophages. **B** H3K4me3 ChIP-seq data for the 869 hub genes defined in **Figure 6** were extracted from **A** and visualized. **C** Mean expression (log2) of the 869 hub genes divided up into the 5 clusters determined by k-mean clustering of all Ensembl genes were visualized in boxplots. **D** Concatenated data for PU.1 from IL4- or IFNγ-stimulated macrophages. **E** PU.1 ChIP-seq data for the 869 hub genes. **F** Mean expression (log2) of the 869 hub genes. Asterisk in **C** reflect statistical significance: * $p < 0.05$, ** $p < 0.01$ (Mann-Whitney U test). Box and whisker plots show the median, 25th and 75th percentile, and the range of expression values for each cluster.

Table Legends to Table S1-S5

Table S1. Gene expression microarray data sample information (related to Figure 1). A Overview of numbers of samples for each cell type and condition. **B** Detailed description of samples included in the study. **C** Explanation of abbrevations for cell stimuli. **D** 9498 unique present genes identified by primary data handling and mean log2-transformed expression values at 29 macrophage conditions

Table S2. 49 modules identified from WGCNA (related to Figure 3). A Pearson correlation coefficients and p-values of eigengenes for 49 modules from 29 conditions. **B** 9498 unique present genes clustered into 49 modules. **C**-**K** Overrepresented pathways, gene ontology and transcription factors in IFNγ-, IL4- and TPP-associated modules identified in InnateDB.

Table S3. Reverse engineering of regulatory networks by ARACNe and TINGe (related to Figure 6). A Parameters and result summary for reverse engineered networks. **B** All genes involved in the ARACNE based network (Bonferroni corrected) and their attributes (degree of connectivity, expression values). **C** Comparison of ranks based on degree of connectivity in networks reverse engineered with ARACNE versus TINGe with same parameter settings. **D** Identification of publications associated with the major hub genes using pubatlas.org. **E** GO-terms revealed by Gene Ontology Enrichment Analysis (GOEA) and visualized using BiNGO. **F** First neighbors of the 5 transcription factors shown in Figure 6D. G Transcription factors identified among the top 10% hub genes, their predicted binding sites at gene loci of the 10% hub genes, and their mean expression (log2) in the 10 clusters identfied in Figure 1I.

Table S4. Comparison of murine macrophage and dendritic cell (DC) signature genes expression across human dataset (related to Figure 7). Fold changes and mean log2-expression values of **A** macrophage signature genes comparing all macrophage endpoint samples against all dendritic cell endpoint samples, **B** dendritic cell signature genes comparing all dendritic cell endpoint samples against all macrophage endpoint samples, **C** macrophage signature genes comparing endpoint samples of each single macrophage condition against all dendritic cell endpoint samples, **D** dendritic cell signature genes comparing all dendritic cell endpoint samples against endpoint samples of each single macrophage condition, **E** macrophage core signature genes comparing endpoint samples of each single macrophage condition against mature dendritic cell endpoint samples, **F** dendritic core signature genes comparing mature dendritic endpoint samples against endpoint samples of each single macrophage condition.

Table S5: Abbreviations and descriptions of used algorithms and software (related to Experimental Procedures).

Supplemental Experimental Procedures

Isolation of human blood-derived cells

Buffy coats from healthy donors were obtained according to protocols accepted by the institutional review board at the University of Bonn (local ethics votes no. 288/13). Written consent was provided for each specimen according to the Declaration of Helsinki. $CD14^+$ monocytes, $CD19^+$ B cells, $CD56^+$ NK cells and $CD3^+$ T-cells were isolated from peripheral blood mononuclear cells (PBMCs) of healthy blood donors using CD14, CD19, CD56 and CD3 Micro Beads (Miltenyi) according to the manufacturer's protocol. Isolated cell fractions were analyzed by flow cytometry to ensure that possible impurities with other leukocytes were lower than 5%. Non-activated T-cells, B-cells, NK-cells, and monocytes were lyzed in Qiazol (Qiagen) immediately after isolation without further cell culture. T-cells and monocytes were activated with different stimuli prior to lysis (**Table S1**). $CD14^+$ monocytes were used for further differentiation into macrophages or dendritic cells.

Human macrophage generation

For *in vitro* differentiation of monocytes into macrophages (M^b, baseline), isolated cells were subsequently cultured in a humidified atmosphere at 37°C, 5% CO_2 for 3 days in RPMI1640 (PAA) supplemented with 10% FCS (Gibco) and 1% PenStrep solution (Gibco) in the presence of 500 IU/ml rhMCSF or 50 IU/ml MCSF. Differentiation into macrophages was analyzed by flow cytometry.

Human macrophage activation

A total of 29 stimuli were used for activation of baseline macrophages (**Figure S2**) inlcuding IFNβ (100 U/ml), high-density lipoprotein (HDL, 2 mg/ml, CSL Behring), IL10 (100 IU/ml), glucocorticoids (GC, dexamethasone, 1 µM, AbZ Pharma), IL4 (1000 IU/ml), IL13 (100 IU/ml), ultrapure lipopolysaccharide (upLPS, 10ng/ml, Sigma), immune complexes (IC, 200µg/ml, Sigma), Pam3CSK4 (P3C, 1 µg/ml, Invivogen), prostaglandine E2 (PGE_2, 1 µg/ml, Sigma), fatty acids (150 µM, all purchased from Sigma, complexed at 65°C with BSA (Sigma)): palmitic acid

(PA), stearic acid (SA), lauric acid (LA), linoleic acid (LiA), or oleic acid (OA), IFNγ (200 IU/ml), TNF (800 IU/ml), standard LPS (sLPS, 10ng/ml, Sigma) or combinations thereof. All cytokines were purchased from Immunotools if not indicated otherwise.

Generation and differentiation of cells by GM-CSF+IL4 (dendritic cells)

Immature dendritic cells (imDCs) were derived from monocytes by 6-day cultures with 800 IU/ml rhGMCSF and 500 IU/ml rhIL4 (Immunotools) in RPMI1640 supplemented with 10% FCS (Gibco) and 1% PenStrep solution (Gibco). For further differentiation of imDC into several subtypes, DC were further stimulated with either rhTNF (800 U/ml, Immunotools) and αCD40 mAbs to induce $CD83^+$ $CD80^+$ DCs, with rhTNF and P3C (1 µg/ml, Invivogen) to induce IDO^+ $CD25^+$ DCs or with upLPS (10 ng/ml, Sigma).

Phenotypic analysis of cells under study

To differentiate between cell subtypes of the myeloid lineage expressions of several surface molecules were analyzed by flow cytometry. Cell suspensions were washed twice in ice-cold FACS buffer (10%FCS in PBS), incubated with each antibody for 30 min and washed subsequently with ice-cold PBS. Following antibodies were used: CD1a, CD1b, CD1c, CD3, CD11b, CD11c, CD13, CD14, CD19, CD23, CD32, CD25, CD56, CD64, CD80, CD86, CD163, CD197, CD206, CD209, CXCR7 (all from BD or BioLegend) and MERTK (R&D Systems). For all antibodies the respective isotype controls were used. Data were acquired on a FACS LSRII cytometer (BD), and analyzed using FlowJo software (Tree Star).

ELISA detection of soluble effector molecules

Levels of soluble CXCL5 (R&D Systems) and IL1a (BioLegend) in the supernatants of M^b, IFNγ-, IL4- and TPP-activated macrophages were measured with ELISA kits in accordance with the manufacturer's instructions.

Western Blot analysis of STAT4 in macrophage subtypes

Protein was extracted by incubating cells with cold lysis buffer containing 1 M TrisHCl pH 8.0, 10% Triton, 1 M NaCl, 0.5 M EDTA, 0.1 M DTT and Protease Inhibitor (Roche Diagnostics) for 30 minutes. Subsequently lysed cells were centrifuged at 13000 rpm on 4°C for 10 minutes. Protein containing supernatant was collected for further analysis. 50 µg of protein samples were fractionated on 10% sodium dodecyl sulphate polyacrylamide gels and transferred onto nitrocellulose membranes (Hyobond-C™ Extra, Amersham Biosiences). Immunoblotting with the BioRad MiniProtean®System was performed at 100mA and 4°C over night. Membranes were blocked with 1xTBST containing 5% of powdered milk (Bio Magermilch Pulver, Heirler Cenovis GmbH) for 60 min. Primary antibodies for STAT4 (sc-486. SantaCruz, dilution 1:1000), and ß-Actin (MAB1501R, Merck Millipore, dilution 1:2500) were incubated over night at 4°C diluted in Blocking Buffer (Li-cor Biosciences). After washing of membranes with 1xTBST membranes were incubated with matching secondary antibodies (IRdye®800CW, Li-cor Biosciences, dilution factor 1:5000 to 1:15000 in 1:1 Blocking Buffer) for 2h at room temperature. Signals were detected on the Odyssey system (Li-cor Biosciences). Band intensity analysis was performed using ImageJ software.

T-cell activation assays in presence of macrophages

Allogenic $CD4^+$ T-cells were isolated via MACS technique, according to the protocol provided by the manufacturer (Miltenyi Biotech). $CD4^+$ T-cells were labeled with carboxyfluorescence in diacetate succinimidyl ester (CFSE) and incubated in 96 well plates with M^b, IFNγ-, IL4- and TPP-activated macrophages at a ratio of 10 to 1 (T-cells: macrophages). Activation of T-cells was achieved with beads coated with anti-CD3 mAb (Janssen-Cilag) or with anti-CD3 and anti-CD28 mAbs at ratios of 1:1 (beads/T-cells). T-cell proliferation was assessed 72h later by flow cytometry. The data were acquired with the LSRII cytometer (BD) and analyzed with the cell proliferation tool of FlowJo (Tree Star).

RNA isolation

RNA was isolated from cells lysed in Qiazol using the miRNeasy Mini Kit (Qiagen) according to the manufactures' protocol. The precipitated RNA was solved in RNAse free water. The quality of the RNA was assessed by measuring the ratio of absorbance at 260 nm and 280 nm using a Nanodrop 1000 Spectrometer (Peqlab) as well as by visualization of the integrity of the 28S and 18S band on a 1.2% agarose gel.

Gene expression profiling by Illumina Beadchip arrays and primary data handling

Prior to array based gene expression profiling total RNA was further purified using the MinElute Reaction Cleanup Kit (Qiagen). Biotin labeled cRNA was generated using the TargetAmp Nano-g Biotin-aRNA Labeling Kit for the Illumina System (Epicentre). Biotin labeled cRNA (1.5 µg) was hybridized to Human HT-12V3 or Human WG-6V3 Beadchips (Illumina) and scanned on an Illumina HiScanSQ system. Raw intensity data were processed in Genome Studio (Illumina) excluding probesets with missing bead types to increase validity. A total of 384 samples were imported into Partek Genomics Suite (PGS) for further analysis including quantile normalization. Batch effects of separate array experiments were removed from normalized log_2-transformed data. Background signal was calculated within R based on coefficient of variation (the computed background for the entire dataset was 6.747). Genes are only kept for further analysis if their mean expression values are higher than background in at least one condition from 299 macrophage transcriptomes. Afterwards, multi-probes were filtered to include only one probe with highest mean expression representing corresponding gene. Only 9,498 unique present genes, which represent most informative genes, were retained for analyses with macrophage activation conditions. They are listed in **Table S1D** with their mean expression values in 29 macrophage conditions).

Bioinformatics to determine the structure within the dataset

We performed co-regulation analysis (CRA), self-organizing map (SOM)-clustering and hierarchical clustering (HC) on correlation coefficient matrices (CCM) to determine the structure within the dataset.

Co-regulation analysis by BioLayout Express^{3D}

BioLayout Express^{3D} (BioLayout) is a powerful tool for the visualization and analysis of network graphs (Theocharidis et al., 2009). We applied BioLayout to distinguish baseline macrophages from other immune cells and to compute and visualize the correlation of 29 different macrophage activation programs. Correlation between macrophages and other immune cells was computed with a Pearson correlation cutoff of 0.96, 0.96, 0.95 and 0.93, respectively (**Figure S1B-E**), and that of macrophage activation programs with a cutoff of 0.95 (**Figure 1B-G**) to maximize the number of samples to be visualized as well as to maximize the distribution of the different categories.

Self-Organizing-Map (SOM) clustering

To reduce the dimensionality of high-dimensional data for visualization, SOM clustering is an excellent tool, which projects the input space on prototypes of a low-dimensional regular grid that can be effectively utilized to explore properties of the data (Kohonen, 1982). SOM clustering was performed to classify the different macrophage activation conditions using PGS. First, the expression values were standardized to a mean of zero and standard deviation of one and this was followed by 20,000 training iterations to cluster similar probes close to each other on the map. In our settings, the whole transcriptome was divided into 10×10 clusters (approximately 400 probes in each cluster), and the expression values of each cluster genes are rescaled to one eigenvalue, which represent the general expression value of this cluster. The resulting data are then visualized as a heatmap representing increased values in red, decreased values in blue and intermediate values in green. It needs to be mentioned that the input data (e.g. number of samples or conditions) influences the cluster structure and standardized mean expression values.

Correlation coefficient matrices combined with hierarchical clustering

To further validate the BioLayout data, we computed Pearson correlation coefficients (PCC) in a pairwise fashion between all macrophage activation conditions using PGS, which results in CCM. PCC was computed using Pearson (Linear) correlation based on expression of the 1000 most variable probes out of 2-way ANOVA based on t-test statistics. We performed hierarchical clustering using Euclidean distance on columns and plotted the standardized correlation coefficient (mean of zero and standard deviation of one) for the macrophage activation conditions. This resulted in 10 larger clusters representing all 29 conditions (**Figure 1I**).

Calculation of the vectors for the spectrum model of macrophage activation

The spectrum model of macrophage activation was established by grouping the samples according to the clusters obtained by the CCM analysis, utilizing the 3D coordinates of the individual macrophage samples determined by CRA, calculating mean vectors for the clusters and plotting the information in a 3D graph using the coordinates of the baseline macrophages (M^b) as the origin. The coordinates of the nodes can be joined by conditions or clusters using 'Collapse Nodes by Class' function in BioLayout by setting baseline macrophages (M^b) as origin (0, 0, 0) and then the joined coordinates of other conditions or clusters are rescaled based on the origin in 3D space. The vectors starting from M^b to all activation states were plotted in 3D using Matlab (**Figure 1J**).

Linking module information to prior knowledge stored in InnateDB

A major resource for innate immunity is the database InnateDB designed to facilitate systems-level analyses of mammalian innate immunity networks, pathways and genes (Breuer et al., 2013). In order to link our experimental data to this knowledge resource we used the WGCNA-defined modules for IFNγ-, IL4- and TPP-induced macrophage activation to analyze the enrichment of pathways, gene ontologies and transcription factor binding sites by InnateDB associated with the three chosen module-associated gene sets. Overrepresentation analyses were performed with standard settings, recommended analysis

algorithms including the hypergeometric statistics and p-value correction method by Benjamini Hochberg. Results are summarized in **Table S2C-K**.

Gene module analysis using WGCNA algorithm

Weighted gene co-expression network analysis (WGCNA) can be used to identify underlying data structures in a complex dataset (Langfelder and Horvath, 2008). We utilized WGCNA to identify co-regulated genes associated with the 29 different macrophage conditions. The WGCNA R package (http://labs.genetics.ucla.edu/horvath/htdocs/CoexpressionNetwork/Rpackages/WGCNA/) was used for the analysis. The standard parameters were altered to a power of 6 and a minModuleSize of 30 resulting in 49 modules using 9498 transcripts in 160 macrophage samples (**Table S2B**). For each module the eigengene corresponding to the first principal component of a given module was calculated. The network for each module of interest was generated using the "1-TOMsimilarityFromExpr" function of the WGCNA R package. The network was subsequently imported into Cytoscape for GO-enrichment analysis and visualization using BiNGO, Enrichment Map, and Word Clouding. Additionally, the 3 most positive correlated modules specific for each condition (IFNγ, IL4 and TPP) were used to visualize a TF correlation network. Genomatix was used to filter module genes for known TFs. A TF coregulation network was calculated using the TFs within the module associated with a particular stimulation condition (e.g. IL4) and using all microarray samples of that particular condition. Visualization of the network was performed in Cytoscape.

Gene ontology enrichment analysis (GOEA) and GO network visualization

To link our data to prior knowledge we performed GOEA by using the Cytoscape (http://www.cytoscape.org/) plug-in BiNGO (v2.44) (Maere et al., 2005). To include only significant results, the FDR threshold was set to 0.05. The Cytoscape plugins Enrichment Map (v1.1) (Merico et al., 2010) and Word Cloud (Oesper et al., 2011) were used to visualize the GO networks. The cutoff for the Jaccard coefficient was set to 0.25 and the FDR q-value to 0.1.

Linking *in vitro* macrophage activation data to *in vivo* macrophage signatures

Using Macrophage.com as a resource, we identified two datasets of human alveolar macrophages (GSE13896 (Shaykhiev et al., 2009) and GSE2125 (Woodruff et al., 2005)) compiling samples from 39 non-smokers, 49 smokers and 12 COPD patients as one dataset. RMA normalization and batch correction was performed by using PGS. Differentially expressed genes were defined by a 1-way ANOVA model (|FC| > 2, FDR (Benjamini and Hochberg, 1995) adjusted p-value < 0.05) to determine differences between macrophages from non-smokers, smokers and COPD patients and used to create co-regulation networks. Gene Set Enrichment Analysis (GSEA) was performed on 49 WGCNA modules from **Figure 3** in 10,000 permutations using PGS (Subramanian et al., 2005). For each comparison (non-smoker versus smoker or COPD patients), normalized enrichment score (NES), allowing comparisons of overrepresentation between different gene sets, together with p-values of GSEA were plotted by Volcano plots. Enriched modules (p-value < 0.01) were selected to perform GOEA.

Linking human macrophage activation to ImmGen macrophage and DC core signatures

To assess the regulation of genes recently defined as core signatures of murine macrophages (Gautier et al., 2012) and dendritic cells we compiled a dataset of 161 macrophages (29 conditions), 33 DCs and 7 monocyte samples (Miller et al., 2012; **Table S1B**). The data set was $\log_2$-transformed, quantile normalized, batch-corrected, and further analyzed using PGS. In order to define the expression of the 44 murine macrophage and 43 murine DC signature genes in the human dataset, the mouse gene symbols were converted to the respective human orthologues by combining information from Ensembl BioMart (http://www.ensembl.org/biomart/martview) using the species specific gene symbols and Ensembl Gene IDs. For some of the genes BioGPS (http://biogps.org/#goto=welcome) was used if no orthologue was found in BioMart. For three genes (A930039A15Rik, H2-Eb2, Klri1) no orthologue could be identified in both resources. For the other genes, the transcript with the highest mean expression across all samples was kept as a representative, where nine genes (Akr1b10, Ap1s3, Btla, H2-Aa, Haao, Hmgn3, Pon3, Pvrl1, Tmem195) were excluded because all corresponding transcripts in the human dataset were not defined as being present. For each transcript having a mean expression higher than the background level

(6.747 on log2 scale) within at least one of the conditions fold changes (FC) were calculated, comparing all macrophages against all DCs. The condition-wise mean expression values were standardized as well as scaled over all examined conditions (to a mean of zero and a standard deviation of one as well as to a minimum value of -2 and a maximum value of 2), sorted according to the overall fold change and visualized in a heatmap using Mayday 2.13 (Battke et al., 2010). Overall fold changes (**Table S4**) were displayed with the heatmap.

Reverse engineering of the core macrophage activation network

The overall approach utilized for reverse network engineering is presented as a schema in **Figure 6A**. Information-theoretic methods such as ARACNe (Basso et al., 2005; Margolin et al., 2006) or TINGe (Aluru et al., 2013) have been introduced to determine central hubs within a dataset. To determine the central hubs of all stimulation conditions reflecting the core macrophage activation network, transcriptional interactions between genes were first determined by ARACNe, which has been integrated into geWorkbench (v2.4.0) (Floratos et al., 2010). For this analysis we used 299 arrays describing all stimulation conditions of macrophage activation. The 9498 unique present genes, as introduced above (**Table S1D**), were taken into calculation of mutual information (a measure of the mutual dependence of the two genes as random variables) with p-value less than 1e-7 with or without Bonferroni correction (**Figure S4A**). The threshold of the data processing inequality (DPI) theorem from information theory used by ARACNe was set to 0.1 and used to detect and discard indirect interactions that are unlikely to be mediated by an actual physical interaction. The functional relationship between the numbers of nodes and their degree of interactions was estimated by power law regression (**Figure S4D**). The power law regression in the relationship between the number of nodes (number of genes ranging from 490 to 1) and their degree (number of interactions ranging from 1 to 164) suggests a scale-free network structure, i.e. the network is unevenly populated with highly connected nodes or hubs and less dense nodes. The R-squared value was 0.767, indicating high correlation and a strong linear relationship between degree of connectivity and corresponding number of genes. Networks were visualized in a force-directed layout in Cytoscape (**Figure 6B** and **Figure S4B**). The plug-in MultiColoredNodes (Warsow et al., 2010) was used to visualize mean expression values of the 10 most highly interconnected genes as well as transcription factors.

Since the introduction of ARACNe several improvements of the original algorithm and novel algorithms have been introduced for reverse network engineering of transcriptome datasets. To ensure robustness of the computed network, we applied a second reverse engineering algorithm, namely the TINGe (Tool for Inferring Network of Genes) algorithm (Aluru et al., 2013). Written in C++ TINGe is also based on information theory. To compare results from ARACNe and TINGe derived networks, we applied the same significance threshold ($p < 1e-7$) and the same DPI tolerance (0.1). For complete workflow see **Figure S4A**.

The inferred networks from ARACNe and TINGe were compared topologically by the degree of connectivity of each gene using a degree-degree plot, where the degrees of ARACNe network genes were in x-axis and corresponding degrees in TINGe network in y-axis (**Figure S4C**). Networks were visualized in a force-directed layout in Cytoscape, followed with statistical analysis such as the functional relationship between the numbers of nodes and their degree of interactions (degree node distribution) of three predicted networks (**Figure S4D-F**) utilizing the plug-in Network Analysis (Cline et al., 2007).

Candidate gene prioritization approach

Reverse engineered networks can predict novel functions for uncharacterized genes but also potential functional associations among known genes. Predictions made by such networks can also be supplemented with prior knowledge or additional data sources to generate new hypotheses for further investigation. We therefore supplemented the network generated by ARACNe or TINGe with prior knowledge by applying the following strategy. The top 10% highly connected hub genes with a degree of connectivity higher than 30 were prioritized by association with macrophage lineage and activation information using the transcription factors PU.1 and RUNX1 as bait genes. Both have crucial roles as macrophage lineage and activation factors. Using these genes we performed similarity profiling, data fusion and network-based strategies by applying two prioritization tools, ToppGene (Chen et al., 2009) and Endeavour (Tranchevent et al., 2008). The results of the different approaches were subsequently combined by the Borda ranking method.

Common transcription factor binding site prediction

TF binding prediction was performed using the Genomatix Suite (http://www.genomatix.de/). First, promoter models for the top 10% hub genes (842 accepted gene loci, 3882 promoter models) of the ARACNe-defined network were compiled using the Gene2Promoter module in Genomatix. Subsequently, the Genomatix module for the search of common TF binding sites was applied to determine overrepresented TF binding sites. Significance measure for each TF family is represented by z-score, calculated with a continuity correction using the formula z = (x-E-0.5)/S, where x is the number of found matches in the input data, E is the expected value and S is the standard deviation. A z-score below -2 or above 2 can be considered to be statistically significant. The z-score was subsequently converted as a normal distribution to the corresponding p-value using pnorm command in R. Data can be found in **Table S3G**.

miRNA-Seq data generation and analysis

Sequencing of miRNAs was done according to the manufacturer's recommendations. In brief, $5x10^6$ -$2x10^7$ macrophages were harvested and total RNA including small RNAs was isolated. Small RNA libraries were generated from 1 µg total RNA with the TruSeq Small RNA Sample Preparation Kit (Illumina). After successful ligation of 3' and 5' adapters to RNA molecules, RNA was reverse-transcribed using SuperScript II reverse transcriptase (Invitrogen). cDNA was amplified by 11 PCR cycles with high-fidelity Phusion Polymerase (Finnzymes). cDNA with the size of miRNAs plus ligated adapters was purified on a pre-cast 6% Tris/Borate/EDTA polyacrylamide gel electrophoresis gel (Invitrogen). Generation of clonal clusters from single molecules of the cDNA library was done with the TruSeq Cluster Kit (Illumina) on a CBot station. Sequencing by synthesis was performed by using the TruSeq SBS Kit on a HiScanSQ system (Illumina). Sequencing reads were retrieved as FASTQ files. After demultiplexing adapter sequences were trimmed from each read using Flicker 3.0 (Illumina). Trimmed reads were mapped to the human genome hg19 and hairpin and mature human miRNAs deposited in miRBase version 19 using the short read aligner Bowtie 0.12.9 (Langmead et al., 2009) with no mismatches allowed. The number of reads mapping to a specific miRNA sequence were counted within PGS. The dataset was then normalized by

using the statistical software R package DESeq (Anders and Huber, 2010) and miRNAs having less than one normalized read count in all samples were excluded. The read counts were transformed into log2 counts per million (cpm) and were divided by the corresponding library size (in millions) by using the R package limma (Smyth, 2005). The R package sva (Johnson et al., 2007) was used to perform a batch removal for the random factors date and donor. Then miRNAs having less than one transformed read count in all samples of the same condition were excluded. Differentially expressed miRNAs between macrophages polarized with IFNγ (M1), IL4 (M2) or with the combination of TNF, PGE_2 and P3C (TPP) were determined against M^b by using the R package limma (Smyth, 2005) with a p-value of 0.05 as well as an absolute fold change of 2 as cutoffs. Finally, for each condition a set of uniquely differentially expressed miRNAs was determined, which was then sorted by the transformed expression values. The first five most highly abundant up-regulated and the first five most highly abundant down-regulated miRNAs were chosen to be represented within a heatmap. Displayed are the fold changes against M^b colored from blue to red.

Histone modification and TF ChIP-Seq data generation and analysis

Native ChIP (N-ChIP) experiments to assess histone modifications were performed following previously described methodology (Cuddapah et al., 2009). Briefly, $2x10^7$ macrophages were harvested from cell cultures and were digested with MNase (0.3U/ml; Sigma Aldrich) to generate mononucleosomes. An additional sonication step was performed three times for 20 seconds each in ice water (Bandelin Sonoplus). 10 µg of rabbit monoclonal anti-trimethyl histone H3K4 antibody (Millipore; 17-614) or purified rabbit IgG were used for each ChIP experiment. For mapping PU.1 binding, ChIP experiments were performed following the manufacturer's protocol (Millipore; Magna ChIP A Kit). In brief, $1x10^7$ macrophages were crosslinked with formaldehyde (1% v/v, 10 min). Next, nuclei were isolated and resuspended in 130 µl Nuclear Lysis Buffer. Chromatin was fragmented to 200-500 bp by ultrasonication in microTUBEs (Covaris) using the Covaris S220 Ultrasonicator (Peak Incident Power 105 (W), Duty Factor 10%, Cycles per Burst 200, treatment time 25 minutes). 10 µg of polyclonal rabbit anti-PU.1 antibody (Santa Cruz Biotechnology; sc-352x) or normal rabbit IgG (Millipore; 12-370) were used. Multiplex DNA libraries of both H3K4me3 as well as PU.1 bound DNA were generated using Illumina's ChIP-Seq Sample Preparation Kit (Illumina; IP-102-1001) and

the Multiplexing Sample Preparation Oligonucleotide Kit (Illumina; PE-400-1001) using at least 10 ng DNA following the manufacturer's instructions. Purified DNA ends were repaired using PNK and Klenow enzyme, followed by treatment with Klenow exo minus polymerase to generate a protruding 3′ A base used for adaptor ligation. Next, size selection of libraries was performed as follows: DNA libraries were agarose gel purified, DNA fragments with approximately 220 bp size excised and eluted using QIAquick Gel Extraction kit (Qiagen). After subsequent adapter ligation to the repaired ends, an amplification step was performed for 5 cycles with PCR primers 1.1 and 2.1 (Illumina, IP-102-1001). During a second amplification step (13 cycles) multiplex PCR primers were added to the DNA libraries to construct multiplex sequencing libraries. For PU.1 DNA libraries multiplex PCR primers were added directly after adapter ligation to the amplification mix and 18 cycles of amplification were performed. Purified DNA was loaded onto a single read SR flowcell (Illumina) and cluster generation performed using the TruSeq SR Cluster Kit on a cBot Cluster station (Illumina). DNA libraries were sequenced with an Illumina HiScan SQ in a multiplex single-read run with at least 40 bases sequencing length and 6 bases for index sequences. Sequence reads from each DNA library were aligned with Casava software (Illumina) against the human reference genome 18 (NCBI 36/hg18) and converted into the .bam file format. To determine permissive histone marks and PU.1 binding sites at major hub gene loci, HM was assessed in M^b, IFNγ- or IL4-stimulated macrophages, or macrophages stimulated with TNF, PGE_2, and P3C (TPP). PU.1 binding was assessed in IFNγ- or IL4-stimulated macrophages. The overall spectrum of histone modifications respectively PU.1 binding was determined by concatenating datasets with Samtools (Li et al., 2009). K-means clustering of concatenated bam files was performed to assess the distribution of marks of interest across the entire gene length with Euclidean distance similarity metric calculations in R as previously described (Statham et al., 2010). For this purpose, average scaled enrichment (ASE) plots were utilized. The number of clusters was set to five. For visualization of H3K4me3 or PU.1 binding the length scaled signal across the entire gene body plus additional 20% upstream and 10% downstream of each gene was calculated. The signal was capped at the 97th percentile of the combined signal from all genes to prevent extreme spikes in enrichment dominating the clustering. For visualization of K-means clustering the Repitools R package was used (Statham et al., 2010).

Active gene regulation in macrophages has been recently linked to open chromatin marks and the presence of the lineage-specific TF PU.1 (Ghisletti et al., 2010; Ostuni et al., 2013). ChIP-seq data of permissive HM H3K4me3 and PU.1 binding sites derived from M^b, M1 (IFNγ), M2 (IL4), and M^{TPP} (TNF+PGE_2+P3C; TPP) were concatenated. HM and PU.1 binding sites were related to the transcriptional start site (TSS). The five major clusters revealed by K-mean clustering of genome-wide information for 37,435 Ensembl genes were visualized by ASE plot (**Figure S5)**. H3K4me3 signals mainly clustering around the TSS, or extending from the TSS into the promoter region and the first exons of the respective gene loci present in all Ensembl genes (**Figure S5A**) where compared with H3K4me3 signals in the 869 major hub genes (**Figure S5B**). Gene expression levels for the major hub genes were plotted according to their H3K4me3 signal cluster affiliation (**Figure S5C**). Instead of H3K4me3, the same analysis was performed for PU.1 binding sites (**Figure S5E-F**).

Relationship analysis of monocyte-derived macrophages (M^b) with other cell types.

As outlined in **Figure 1A and Figure S1A**, macrophages (M^b, baseline) were generated from blood-derived $CD14^+$ monocytes by either stimulating them with rhGMCSF; rhMCSF (n=275) or M-CSF (n=24) and compared to other immune cells including monocyte-derived DCs, further maturated DCs, T-cells, B-cells and NK-cells (**Table S1, n=384**). For comparison, we used co-regulation analysis of transcriptome data (**Figure S1B-E**). Differences between GM-CSF-induced and M-CSF-induced macrophages as previously described (Bailey et al., 2011; Lacey et al., 2012) were assessed. Furthermore, macrophages were compared with $CD14^+$ monocytes, GM-CSF+IL4 stimulated monocyte-derived immature DCs, differentiated DCs ($CD83^+$ DCs, $CD25^+$ DCs, DCs stimulated with upLPS), T-, B-, and NK-cells. Transcriptome data were substantiated by flow cytometry (**Figure S1F, G**).

Link to specialized online resource on human macrophage activation

For this resource dataset of human macrophage activation, we have established an additional web resource that can be reached at the following web address: http://www.macrophages.uni-bonn.de. On this web resource tables with normalized data of the complete dataset, ANOVA-model based comparisons of different conditions, scripts and

software tools used in the study as well as additional information can be found and used for own research purposes.

Tables available on http://www.macrophages.uni-bonn.de

- matrix_unnormalized.txt: gene expression matrix of log2 transformed unnormalized data
- matrix_normalized.txt: gene expression matrix of log2 transformed quantile normalized and batch corrected data
- annotation of HumanHT12v3 arrays.csv: annotation file of HumanHT12v3 array type for matrix data
- ANOVA-baseline vs. other 28 conditions.txt: ANOVA model comparing baseline macrophages with other 28 in vitro conditions
- ANOVA-IFNb vs. other 28 conditions.txt: ANOVA model comparing IFNβ-stimulated macrophages with other 28 in vitro conditions
- ANOVA-HDL vs. other 28 conditions.txt: ANOVA model comparing HDL-stimulated macrophages with other 28 in vitro conditions
- ANOVA-IL10 vs. other 28 conditions.txt: ANOVA model comparing IL10-stimulated macrophages with other 28 in vitro conditions
- ANOVA-GC vs. other 28 conditions.txt: ANOVA model comparing GC-stimulated macrophages with other 28 in vitro conditions
- ANOVA-IL4 vs. other 28 conditions.txt: ANOVA model comparing IL4-stimulated macrophages with other 28 in vitro conditions
- ANOVA-IL13 vs. other 28 conditions.txt: ANOVA model comparing IL13-stimulated macrophages with other 28 in vitro conditions
- ANOVA-IL4_upLPS vs. other 28 conditions.txt: ANOVA model comparing IL4+upLPS-stimulated macrophages with other 28 in vitro conditions
- ANOVA- upLPS_IC vs. other 28 conditions.txt: ANOVA model comparing upLPS+IC -stimulated macrophages with other 28 in vitro conditions
- ANOVA- upLPS vs. other 28 conditions.txt: ANOVA model comparing upLPS-stimulated macrophages with other 28 in vitro conditions
- ANOVA-P3C_PGE2 vs. other 28 conditions.txt: ANOVA model comparing P3C+PGE_2 -stimulated macrophages with other 28 in vitro conditions
- ANOVA- P3C vs. other 28 conditions.txt: ANOVA model comparing P3C-stimulated macrophages with other 28 in vitro conditions
- ANOVA- PGE2 vs. other 28 conditions.txt: ANOVA model comparing PGE_2-stimulated macrophages with other 28 in vitro conditions
- ANOVA-LA vs. other 28 conditions.txt: ANOVA model comparing LA-stimulated macrophages with other 28 in vitro conditions
- ANOVA-OA vs. other 28 conditions.txt: ANOVA model comparing OA-stimulated macrophages with other 28 in vitro conditions
- ANOVA-LiA vs. other 28 conditions.txt: ANOVA model comparing LiA-stimulated macrophages with other 28 in vitro conditions
- ANOVA-SA vs. other 28 conditions.txt: ANOVA model comparing SA-stimulated macrophages with other 28 in vitro conditions

- ANOVA-PA vs. other 28 conditions.txt: ANOVA model comparing PA-stimulated macrophages with other 28 in vitro conditions
- ANOVA-IFNg vs. other 28 conditions.txt: ANOVA model comparing IFNγ-stimulated macrophages with other 28 in vitro conditions
- ANOVA-TNF vs. other 28 conditions.txt: ANOVA model comparing TNF-stimulated macrophages with other 28 in vitro conditions
- ANOVA-IFNg_TNF vs. other 28 conditions.txt: ANOVA model comparing IFNγ+TNF -stimulated macrophages with other 28 in vitro conditions
- ANOVA-sLPS vs. other 28 conditions.txt: ANOVA model comparing sLPS-stimulated macrophages with other 28 in vitro conditions
- ANOVA-sLPS_IFNg vs. other 28 conditions.txt: ANOVA model comparing sLPS+IFNγ-stimulated macrophages with other 28 in vitro conditions
- ANOVA-sLPS_IC vs. other 28 conditions.txt: ANOVA model comparing sLPS+IC -stimulated macrophages with other 28 in vitro conditions
- ANOVA-TNF_PGE2 vs. other 28 conditions.txt: ANOVA model comparing TNF+PGE_2 -stimulated macrophages with other 28 in vitro conditions
- ANOVA-TNF_P3C vs. other 28 conditions.txt: ANOVA model comparing TNF+P3C-stimulated macrophages with other 28 in vitro conditions
- ANOVA-TPP vs. other 28 conditions.txt: ANOVA model comparing TNF+ P3C+PGE_2 (TPP)-stimulated macrophages with other 28 in vitro conditions
- ANOVA-TPP_IFNb vs. other 28 conditions.txt: ANOVA model comparing TPP+IFNβ-stimulated macrophages with other 28 in vitro conditions
- ANOVA-TPP_IFNb_IFNg vs. other 28 conditions.txt: ANOVA model comparing TPP+IFNβ+IFNγ-stimulated macrophages with other 28 in vitro conditions

Supplemental References

Aluru, M., Zola, J., Nettleton, D., and Aluru, S. (2013). Reverse engineering and analysis of large genome-scale gene networks. Nucleic acids research *41*, e24.

Anders, S., and Huber, W. (2010). Differential expression analysis for sequence count data. Genome biology *11*, R106.

Bailey, M.J., Lacey, D.C., de Kok, B.V., Veith, P.D., Reynolds, E.C., and Hamilton, J.A. (2011). Extracellular proteomes of M-CSF (CSF-1) and GM-CSF-dependent macrophages. Immunology and cell biology *89*, 283-293.

Basso, K., Margolin, A.A., Stolovitzky, G., Klein, U., Dalla-Favera, R., and Califano, A. (2005). Reverse engineering of regulatory networks in human B cells. Nature genetics *37*, 382-390.

Battke, F., Symons, S., and Nieselt, K. (2010). Mayday--integrative analytics for expression data. Bmc Bioinformatics *11*, 121.

Benjamini, Y., and Hochberg, Y. (1995). Controlling the False Discovery Rate - a Practical and Powerful Approach to Multiple Testing. J Roy Stat Soc B Met *57*, 289-300.

Breuer, K., Foroushani, A.K., Laird, M.R., Chen, C., Sribnaia, A., Lo, R., Winsor, G.L., Hancock, R.E., Brinkman, F.S., and Lynn, D.J. (2013). InnateDB: systems biology of innate immunity and beyond--recent updates and continuing curation. Nucleic acids research *41*, D1228-1233.

Chen, J., Bardes, E.E., Aronow, B.J., and Jegga, A.G. (2009). ToppGene Suite for gene list enrichment analysis and candidate gene prioritization. Nucleic Acids Res *37*, W305-311.

Cline, M.S., Smoot, M., Cerami, E., Kuchinsky, A., Landys, N., Workman, C., Christmas, R., Avila-Campilo, I., Creech, M., Gross, B.*, et al.* (2007). Integration of biological networks and gene expression data using Cytoscape. Nature protocols *2*, 2366-2382.

Cuddapah, S., Barski, A., Cui, K., Schones, D.E., Wang, Z., Wei, G., and Zhao, K. (2009). Native chromatin preparation and Illumina/Solexa library construction. Cold Spring Harbor protocols *2009*, pdb prot5237.

Floratos, A., Smith, K., Ji, Z., Watkinson, J., and Califano, A. (2010). geWorkbench: an open source platform for integrative genomics. Bioinformatics *26*, 1779-1780.

Gautier, E.L., Shay, T., Miller, J., Greter, M., Jakubzick, C., Ivanov, S., Helft, J., Chow, A., Elpek, K.G., Gordonov, S.*, et al.* (2012). Gene-expression profiles and transcriptional regulatory pathways that underlie the identity and diversity of mouse tissue macrophages. Nature immunology *13*, 1118-1128.

Ghisletti, S., Barozzi, I., Mietton, F., Polletti, S., De Santa, F., Venturini, E., Gregory, L., Lonie, L., Chew, A., Wei, C.L.*, et al.* (2010). Identification and characterization of enhancers controlling the inflammatory gene expression program in macrophages. Immunity *32*, 317-328.

Johnson, W.E., Li, C., and Rabinovic, A. (2007). Adjusting batch effects in microarray expression data using empirical Bayes methods. Biostatistics *8*, 118-127.

Kohonen, T. (1982). Self-Organized Formation of Topologically Correct Feature Maps. Biol Cybern *43*, 59-69.

Lacey, D.C., Achuthan, A., Fleetwood, A.J., Dinh, H., Roiniotis, J., Scholz, G.M., Chang, M.W., Beckman, S.K., Cook, A.D., and Hamilton, J.A. (2012). Defining GM-CSF- and macrophage-CSF-dependent macrophage responses by in vitro models. J Immunol *188*, 5752-5765.

Langfelder, P., and Horvath, S. (2008). WGCNA: an R package for weighted correlation network analysis. Bmc Bioinformatics *9*.

Langmead, B., Trapnell, C., Pop, M., and Salzberg, S.L. (2009). Ultrafast and memory-efficient alignment of short DNA sequences to the human genome. Genome biology *10*, R25.

Li, H., Handsaker, B., Wysoker, A., Fennell, T., Ruan, J., Homer, N., Marth, G., Abecasis, G., Durbin, R., and Proc, G.P.D. (2009). The Sequence Alignment/Map format and SAMtools. Bioinformatics *25*, 2078-2079.

Maere, S., Heymans, K., and Kuiper, M. (2005). BiNGO: a Cytoscape plugin to assess overrepresentation of gene ontology categories in biological networks. Bioinformatics *21*, 3448-3449.

Margolin, A.A., Wang, K., Lim, W.K., Kustagi, M., Nemenman, I., and Califano, A. (2006). Reverse engineering cellular networks. Nature protocols *1*, 662-671.

Merico, D., Isserlin, R., Stueker, O., Emili, A., and Bader, G.D. (2010). Enrichment map: a network-based method for gene-set enrichment visualization and interpretation. PloS one *5*, e13984.
Oesper, L., Merico, D., Isserlin, R., and Bader, G.D. (2011). WordCloud: a Cytoscape plugin to create a visual semantic summary of networks. Source code for biology and medicine *6*, 7.
Ostuni, R., Piccolo, V., Barozzi, I., Polletti, S., Termanini, A., Bonifacio, S., Curina, A., Prosperini, E., Ghisletti, S., and Natoli, G. (2013). Latent enhancers activated by stimulation in differentiated cells. Cell *152*, 157-171.
Shaykhiev, R., Krause, A., Salit, J., Strulovici-Barel, Y., Harvey, B.G., O'Connor, T.P., and Crystal, R.G. (2009). Smoking-dependent reprogramming of alveolar macrophage polarization: implication for pathogenesis of chronic obstructive pulmonary disease. J Immunol *183*, 2867-2883.
Smyth, G.K. (2005). limma: Linear Models for Microarray Data. In Bioinformatics and Computational Biology Solutions Using R and Bioconductor, R. Gentleman, V. Carey, W. Huber, R. Irizarry, and S. Dudoit, eds. (Springer New York), pp. 397-420.
Statham, A.L., Strbenac, D., Coolen, M.W., Stirzaker, C., Clark, S.J., and Robinson, M.D. (2010). Repitools: an R package for the analysis of enrichment-based epigenomic data. Bioinformatics *26*, 1662-1663.
Subramanian, A., Tamayo, P., Mootha, V.K., Mukherjee, S., Ebert, B.L., Gillette, M.A., Paulovich, A., Pomeroy, S.L., Golub, T.R., Lander, E.S., and Mesirov, J.P. (2005). Gene set enrichment analysis: a knowledge-based approach for interpreting genome-wide expression profiles. Proceedings of the National Academy of Sciences of the United States of America *102*, 15545-15550.
Theocharidis, A., van Dongen, S., Enright, A.J., and Freeman, T.C. (2009). Network visualization and analysis of gene expression data using BioLayout Express(3D). Nature protocols *4*, 1535-1550.
Tranchevent, L.C., Barriot, R., Yu, S., Van Vooren, S., Van Loo, P., Coessens, B., De Moor, B., Aerts, S., and Moreau, Y. (2008). ENDEAVOUR update: a web resource for gene prioritization in multiple species. Nucleic acids research *36*, W377-384.
Warsow, G., Greber, B., Falk, S.S.I., Harder, C., Siatkowski, M., Schordan, S., Som, A., Endlich, N., Scholer, H., Repsilber, D., *et al.* (2010). ExprEssence - Revealing the essence of differential experimental data in the context of an interaction/regulation net-work. Bmc Syst Biol *4*.
Woodruff, P.G., Koth, L.L., Yang, Y.H., Rodriguez, M.W., Favoreto, S., Dolganov, G.M., Paquet, A.C., and Erle, D.J. (2005). A distinctive alveolar macrophage activation state induced by cigarette smoking. American journal of respiratory and critical care medicine *172*, 1383-1392.
