# Supplementary figures and images for: Transcriptome-Based Network Analysis Reveals a Spectrum Model of Human Macrophage Activation
Source: Immunity. 2014 Feb 20;40(2):274–88. doi: 10.1016/j.immuni.2014.01.006 (PMC3991396; doi:10.1016/j.immuni.2014.01.006)

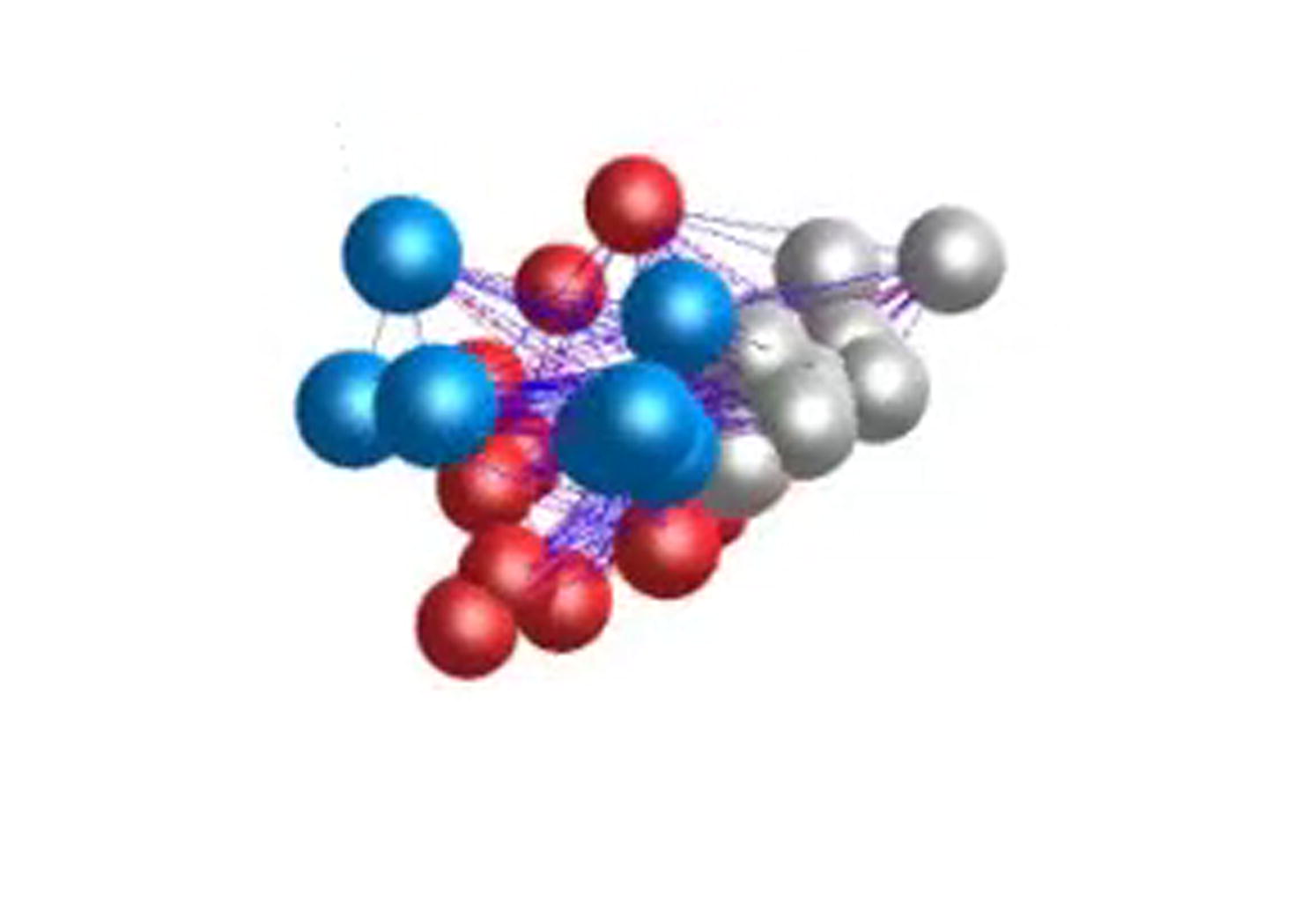

Supplement: Movie S1. Classical M1 versus M2 Polarization — Rotation of coregulation networks with 3, 17, and 29 in vitro conditions in 3-dimensional space (related to Figures 1B, 1E, and 1F). [file mmc7.jpg]
